# Epimutations mimic genomic mutations of *DNMT3A* in acute myeloid leukemia

**DOI:** 10.1038/leu.2013.362

**Published:** 2013-12-20

**Authors:** E Jost, Q Lin, C I Weidner, S Wilop, M Hoffmann, T Walenda, M Schemionek, O Herrmann, M Zenke, T H Brümmendorf, S Koschmieder, W Wagner

**Affiliations:** 1 Department of Oncology, Hematology and Stem Cell Transplantation, RWTH Aachen University Medical School, Aachen, Germany; 2 Institute for Biomedical Engineering—Cell Biology, RWTH Aachen University Medical School, Aachen, Germany; 3 Helmholtz-Institute for Biomedical Engineering, Stem Cell Biology and Cellular Engineering, RWTH Aachen University Medical School, Aachen, Germany

**Keywords:** acute myeloid leukemia, *de novo* methyltransferase DNMT3A, mutation, epimutation, DNA methylation, epigenomics

## Abstract

Mutations in the genetic sequence of the DNA *de novo* methyltransferase *DNMT3A* (DNA methyltransferase 3A) are found in many patients with acute myeloid leukemia (AML). They lead to dysfunction of DNMT3A protein and represent a marker for poor prognosis. Effects of genetic mutations can be mimicked by epigenetic modifications in the DNA methylation (DNAm) pattern. Using DNAm profiles of the Cancer Genome Atlas Research Network (TCGA), we identified aberrant hypermethylation at an internal promoter region of *DNMT3A*, which occurred in about 40% of AML patients. Bisulfite pyrosequencing assays designed for this genomic region validated hypermethylation specifically in a subset of our AML samples. High DNAm levels at this site are particularly observed in samples without genetic mutations in *DNMT3A*. Epimutations and mutations of *DNMT3A* were associated with related gene expression changes such as upregulation of the homeobox genes in *HOXA* and *HOXB* clusters. Furthermore, epimutations in *DNMT3A* were enriched in patients with poor or intermediate cytogenetic risk, and in patients with shorter event-free survival and overall survival (OS). Taken together, aberrant DNA hypermethylation within the *DNMT3A* gene, in analogy to *DNMT3A* mutations, is frequently observed in AML and both modifications seem to be useful for risk stratification or choice of therapeutic regimen.

## Introduction

DNA methylation (DNAm) of CpG dinucleotides is a key epigenetic process. Upon cell division, the DNAm pattern is maintained on the newly synthesized DNA strand, particularly by DNA methyltransferase 1 (DNMT1), whereas DNMT3A and DNMT3B act as *de novo* methyltransferases.^1^ In addition, a coregulatory methyltransferase-like protein, DNMT3L, modulates activity and targeting of DNMT3A and DNMT3B.^2, 3^ Interestingly, all three catalytically active DNMTs are subject to extensive tissue- or developmental stage-specific alternative splicing. Different variants of DNMT3s, with different interaction and binding properties, are even coexpressed within the same cell.^4^ Such alternatively spliced forms of DNMTs may process altered enzymatic activity and they may favor specific genomic regions.^5, 6^

DNAm has a central role in normal hematopoietic development and there is a growing perception that it is also crucial for dysfunctional hematopoiesis in myeloid neoplasms. Conditional ablation of DNMT3A in mice has been shown to increase the stem cell pool and to impair differentiation.^7^ The relevance of DNMT3A is further supported by frequent mutations in acute myeloid leukemia (AML)^8, 9^ and myelodysplastic syndromes.^10^ About 22% of AML patients harbor mutations in *DNMT3A*, either at the highly recurrent position R882 or at other sites within the gene.^8^ Yan *et al.*^11^ have recently reported that mutations in *DNMT3A* are associated with alterations of DNAm and gene expression profiles (such as *HOXB* genes). Furthermore, mutations in *DNMT3A* are associated with a poor prognosis in AML and have hence been suggested to be taken into consideration for risk stratification.^8, 12^ The role of DNMT inhibitors in the treatment of myelodysplastic syndromes and AML has been addressed in multiple clinical trials: treatment with azacytidine resulted in a longer overall survival (OS), a lower transfusion frequency and a delay of transformation from myelodysplastic syndromes into AML. However, the exact mode of action of DNMT inhibitors, predictive biomarkers, best schedule of administration and combination with other treatment modalities is still unknown.^13^

Although it has been recognized that alternative isoforms of DNMT3s may be relevant in AML, this has not been systematically addressed. Very little is known how DNAm is affected within the *DNMT3A* gene—such modifications may be involved in the regulation of alternative isoforms.^14, 15^ We hypothesized that epigenetic modification within the gene *DNMT3A* might affect the expression of variant transcripts and thereby mimic mutations in *DNMT3A*. To this end, we have analyzed DNAm profiles of various publically available data sets based on the Illumina HumanMethylation450K BeadChip. This platform assays more than 480 000 CpG sites at single base resolution (covering 99% of RefSeq genes and 96% of CpG islands (CGIs)).^16^ We demonstrate that DNAm profiles in AML reveal frequent hypermethylation at an internal promoter region of *DNMT3A*, which was not observed in normal blood. Furthermore, we provide evidence that this aberrant hypermethylation might be associated with a similar molecular sequel and clinical consequences as genomic mutations in *DNMT3A*.

## Materials and methods

### Blood samples

Blood samples of healthy donors and patients were taken after written consent according to the ‘Biobank' rules of the medical faculty of the University of Aachen (Permit Number: EK206/09) and the study has been specifically approved by the local ethics committee. Diagnosis of AML or myeloproliferative neoplasms was established according to the World Health Organization criteria and patient characteristics are summarized in Supplementary Table 1. For additional control, stem cell harvests obtained by leukapheresis from patients with solid tumors or non-Hodgkin lymphoma without evidence of blood infiltration were analyzed (EK206/09).

### DNAm analysis by pyrosequencing

Genomic DNA was isolated using the QIAamp DNA Blood Mini Kit (Qiagen, Hilden, Germany). One microgram of DNA was sodium bisulfite modified using the EZ DNA Methylation Kit (Zymo Research, Irvine, CA, USA). The region of interest was amplified by polymerase chain reaction (PCR) using the first primer pair (Supplementary Table 2). A single-strand linear DNA was prepared from the PCR product with the PyroMark TM Vacuum Prep Workstation (Qiagen). The sequencing PCR was then performed with a gene-specific sequencing primer on a PyroMark Q96 ID System (Qiagen, Hilden, Germany) and analyzed with PyroMark Q CpG software (Qiagen).

### Sequencing of *DNMT3A* exons 18, 19 and 23

Mutational analysis was performed for the *DNMT3A* hotspot region in exon 23, and for the frequently mutated exons 18 and 19 (NM_175629.2). Fifty nanograms of DNA were amplified using PCR primers that had been described before.^17^ The PCR product was purified using QIAquick PCR Purification Kit (Qiagen) and Sanger sequencing was performed at MWG (Louisville, KY, USA).

### qRT-PCR analysis of variant transcripts

Expression of *DNMT3A* transcripts was analyzed by quantitative real-time PCR (qRT-PCR) using the StepOne Instrument (Applied Biosystems, Darmstadt, Germany). RNA was isolated using the miRNeasy Mini Kit (Qiagen). Quality control and measurement of RNA concentration was carried out with a NanoDrop Spectrophotometer (Thermo Scientific, Wilmington, DE, USA). Total RNA (1 μg) was reverse-transcribed using the high-capacity cDNA Reverse Transcription Kit (Applied Biosystems) and amplified using FAST SYBR Green PCR Master Mix (Applied Biosystems). Because of differential expression of the housekeeping gene (*GAPDH*) in AML *versus* healthy control samples (data not presented) and high interindividual variation of *DNMT3A* expression, we have normalized expression of either transcript 2 or 4 towards the expression of *DNMT3A* transcripts 1 and 3 (which cannot be distinguished by the primers; Supplementary Table 3).

### Analysis of DNAm profiles

For this study, we used publically available data sets of DNAm profiles generated with the HumanMethylation450K BeadChip platform: GSE35069;^18^ GSE40699 (Encode project); GSE40279;^19^ and TCGA (https://tcga-data.nci.nih.gov/tcga/). *β*-Values ranging from 0 (non-methylated) to 1 (100% methylation) are provided for each CpG site. The 450K BeadChip comprises 79 CpGs related to the gene *DNMT3A*. However, in this study we focused on 72 CpGs with *β*-values in all TCGA samples (missing values might be due to low bead numbers) and these were also considered for graphical presentation. For statistical analysis, we categorized samples as indicated. Raw *P*-values were then calculated using limma *t*-test in R/Bioconductor and further adjusted by the Benjamini–Hochberg method. CpG sites with adjusted *P*<0.05 and an additional cutoff of 20% DNAm change were considered as being differentially methylated. Affiliation of CpG sites with gene regions or CGIs was used as described in detail before^20^ and according to UCSC Genome Browser (GRCh37/hg19).

### Analysis of gene expression profiles

RNA-sequencing data (UNC IlluminaHiSeq_RNASeqV2; *n*=173) of exon, transcript level and gene level were downloaded from TCGA (https://tcga-data.nci.nih.gov/tcga/). Microarray data (Affymetrix HG-U133_Plus_2; *n*=183) were also downloaded from TCGA and normalized by RMA in R/Bioconductor. Differentially expressed genes were selected by adjusted *P*-values using limma *t*-test (*P*<0.05) with an additional cutoff for two-fold differential gene expression. Hierarchical clustering (Euclidian distance), principal component analysis and heatmaps were calculated in R. Gene ontology analysis and chemical and genetic perturbations were analysed using the Gene Set Enrichment Analysis tool (http://www.broadinstitute.org/gsea/index.jsp). For analysis of relative expression of different transcripts, we first applied quantile normalization to account for high variation between different transcripts and then normalized their expression to transcript 1.

### Biostatistics on clinical parameters

Survival data were analyzed using the Kaplan–Meier method and compared using the log-rank test as well as using the multivariate Cox proportional hazard method. Statistical tests were two-sided and were performed using the SAS software package version 9.1.3 (SAS Institute Inc., Cary, NC, USA). For all tests, *P*<0.05 was used as the level of significance.

## Results

### AML samples reveal differentially methylated regions in *DNMT3A*

**Figure 1:**
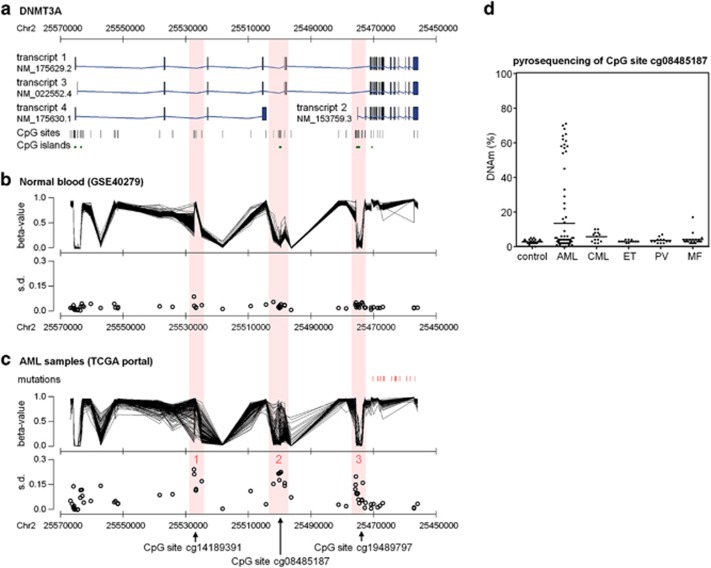
Variable DNAm within *DNMT3A* in AML. (**a**) Schematic presentation of four different transcripts for *DNMT3A.* The positions of corresponding CpG probes represented on the HumanMethylation450K BeadChip platform and their relation to CGIs are depicted. (**b**) *β*-Values (DNAm level) for each of these CpG sites were analyzed in 656 normal blood samples.^19^ Standard deviation (s.d.) of *β*-values revealed relatively little interindividual variation at all CpG sites. (**c**) However, DNAm profiles of 194 AML samples^9^ revealed three DMRs that are highlighted in red. Probe set IDs for the most prominent CpG sites are indicated by arrows. Furthermore, locations of genomic mutations are indicated. Notably, all mutations in *DNMT3A* are related to exons of transcript 2. (**d**) DNAm at the CpG site cg08485187 (within DMR2) was subsequently analyzed by bisulfite pyrosequencing in peripheral blood of healthy controls and patients with AML, CML, essential thrombocythemia (ET), polycythemia vera (PV) and myelofibrosis (MF).

The gene *DNMT3A* comprises 23 exons and four main transcripts have been described (Figure 1a). A large data set of Hannum and co-workers^19^ with 656 blood samples of healthy donors revealed little interindividual variation in the DNAm pattern of CpG sites related to *DNMT3A* (Figure 1b). In contrast, bone marrow-derived blood samples of 194 AML patients from TCGA repository data^9^ revealed three differentially methylated regions (DMRs) within *DNMT3A* (Figure 1c): in comparison with normal blood, DMR1 was hypomethylated in some AMLs. This hypomethylation revealed relatively little overlap with DNA hypermethylation that occurred at DMR2 and DMR3 (Supplementary Figure 1). Hypermethylation at DMR2 and DMR3 was also observed in malignant hematopoietic cell lines, whereas different subsets of mature hematopoietic cells displayed relatively little variation, indicating that DNAm changes are not due to changes in cellular composition (Supplementary Figure 2).^18^ DMR2 and DMR3 comprised each a CGI (Figure 1a). Notably, they correspond to an upstream promoter region of transcript 2, which we have recently identified in hematopoietic stem and progenitor cells: particularly DMR2 was non-methylated in CD34^+^ cells from cord blood and revealed some of the most significant hypermethylation upon culture expansion *in vitro* (*P*<10^−46^).^21^ This led us to the assumption that this region is of particular relevance for maintenance of normal hematopoietic integrity and therefore we focused on DNAm at DMR2.

Bisulfite pyrosequencing assays were designed to address specifically DNAm at DMR2 in blood of healthy controls (*n*=26) and patients with chronic myeloid leukemia (*n*=15), essential thrombocythemia (*n*=13), polycythemia vera (*n*=12), myelofibrosis (*n*=20) and AML (*n*=88; Supplementary Figure 3). As expected, the DNAm level was always below 10% in controls, whereas it was increased in many patients with myeloproliferative neoplasms. Very high DNAm levels at DMR2 were exclusively found in a subset of AML samples (Figure 1d). Furthermore, it was observed in various cell lines derived from AML (KCL-22, K-562, KYO-1, HEL, MV-4-11, KG-1a and HL-60; Supplementary Figure 4). DNA hypermethylation was not restricted to individual CpG sites, but seemed to affect the whole CGI and shore region accordingly (Supplementary Figure 5). Overall, there was no clear association of DNAm at DMR2 with gender and age (Supplementary Figure 6). We defined epimutations to be present if the DNAm level at the CpG site related to probe set cg08485187 was above 10% as this level has not been reached in any of the controls. Notably, 15% of AML samples even revealed a DNAm level of more than 50%, indicating that the epimutation affected both alleles—particularly given that not all cells derive from the malignant clone. When we analyzed DNAm at cg08485187 in relation to the percentage of blast cells, it was evident that there was no linear relationship between blast counts and DNAm at DMR2 (Supplementary Figure 7). This supported the notion that AML samples can be discerned in those with or without epimutation in *DNMT3A*.

### Hypermethylation in DMR2 is higher in samples without genetic mutation in *DNMT3A*

**Figure 2:**
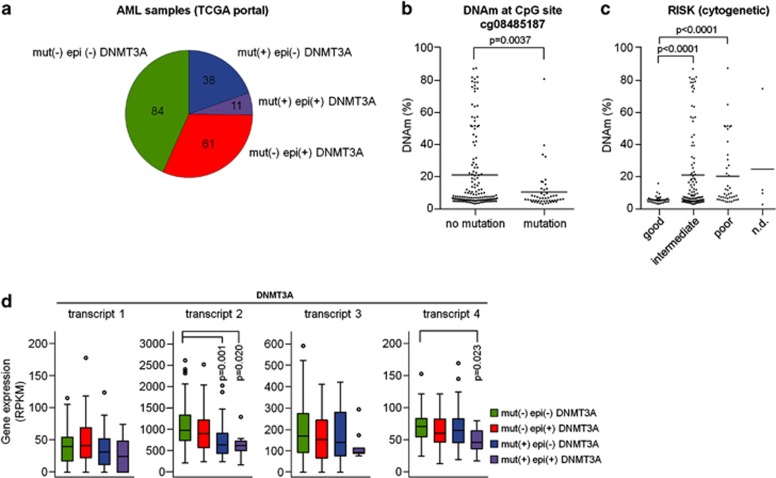
Interplay of epimutations and mutations in *DNMT3A*. (**a**) DNAm profiles of TCGA^9^ were categorized in samples with *DNMT3A* mutation (mut+) and those with aberrant DNA hypermethylation at cg08485187 (*β*-value >0.1), which has been classified as epimutation (epi+). (**b** and **c**) Aberrant DNA hypermethylation was significantly lower in AML samples with *DNMT3*A mutations and it was hardly associated with a good cytogenetic risk score (ND=not determined). (**d**) Analysis of RNA-sequencing data^9^ revealed that *DNMT3A* transcript 2 is slightly less expressed in samples with epimutation and significantly downregulated in samples with *DNMT3A* mutations. Statistical significance was estimated by Wilcoxon's rank-sum test. RPKM=reads per kilobase per million reads.

In several other tumor-associated genes, such as *BRCA1* in ovarian cancer^22^ and *CDKN2A* in squamous carcinoma,^23^ the causal relevance of epigenetic changes has been supported by the finding that such silencing events are mutually exclusive with mutational inactivation of the same gene.^24^ To analyze if DNA hypermethylation at DMR2 is related to genomic mutations of *DNMT3A*, we sequenced the exons 18, 19 and 23 in our AML samples. Three patients had mutations at the hotspot Arg882, one patient revealed a novel mutation at Asn879 and one patient had a stop mutation at Cys710. With our assays, we found genetic mutations exclusively in patients without epimutations, but this was not significant because of the relatively low sample number and because of the restriction of mutational analysis to exons 18, 19 and 23 (Supplementary Figure 8). However, the trend was also observed in the 194 AML samples of TCGA, which provided sequencing information of the entire *DNMT3A* gene:^9^ epimutations and mutations in *DNMT3A* appeared to be vastly mutually exclusive (*P*=0.015, two-sided Fisher's exact test; Figure 2a). Furthermore, the DNAm levels within DMR2 of *DNMT3A* were significantly higher in samples without genetic mutation (*P*=0.0037, Wilcoxon's rank-sum test; Figure 2b). Patients harboring mutations were enriched in the cohorts with intermediate or poor cytogenetic risk as described before (*P*<10^−5^; two-sided Fisher's exact test).^9^ Interestingly, patients with epimutations in *DNMT3A*—even in the subset without mutations—revealed the same enrichment in intermediate- or poor cytogenetic-risk groups (*P*<0.0002; Figure 2c) and the same tendency was also observed in our patients (Supplementary Figure 8d). In contrast, aberrant hypomethylation at DMR1 was associated with favorable cytogenetic risk (Supplementary Figure 9). In analogy to *DNMT3A* mutations, the epimutations were not found in core-binding factor-AMLs (defined by *RUNX1-RUNX1T1* or *CBFB-MYH11* fusion) and only very rarely in acute promyelocytic leukemia (*PML-PARA* fusion).^25^ On the other hand, mutations as well as epimutations in *DNMT3A* were highly significantly associated with mutations of *IDH1*, *IDH2*, *RUNX1* and *NPM1* (Supplementary Figure 10).

### Epimutations and mutations in *DNMT3A* downregulate relative expression of *DNMT3A* transcript 2

Hypermethylation at DMR2 and DMR3, which seem to be related to the internal promoter region of transcript 2, may result in the downregulation of *DNMT3A* transcript 2. In fact, analysis of RNA-sequencing data of TCGA^9^ revealed that particularly those exons included in transcript 2 were highly expressed in AML samples (Figure 2d). Interestingly, they were slightly less expressed in patients with epimutation (Supplementary Figure 11a). Because of the high interindividual variation in *DNMT3A* expression, we have determined relative expression as compared with transcript 1. In fact, transcript 2 was then slightly less expressed in patients with epimutation in *DNMT3A* (*P*=0.026) and the same tendency was also observed in our AML samples using quantitative real-time PCR (qRT-PCR) (Supplementary Figures 11c–e). Unexpectedly, an even more pronounced downregulation of transcript 2 was observed in patients with mutations in *DNMT3A*, even though the frequently mutated exon 23 is usually also part of transcripts 1 and 3 (Figure 2d). In this regard, it is also interesting that all genomic mutations in *DNMT3A* described in TCGA data were associated with exons of transcript 2, but not with DMR2 or DMR3 directly (Figure 1c).

### DNAm changes associated with *DNMT3A* epimutations

**Figure 3:**
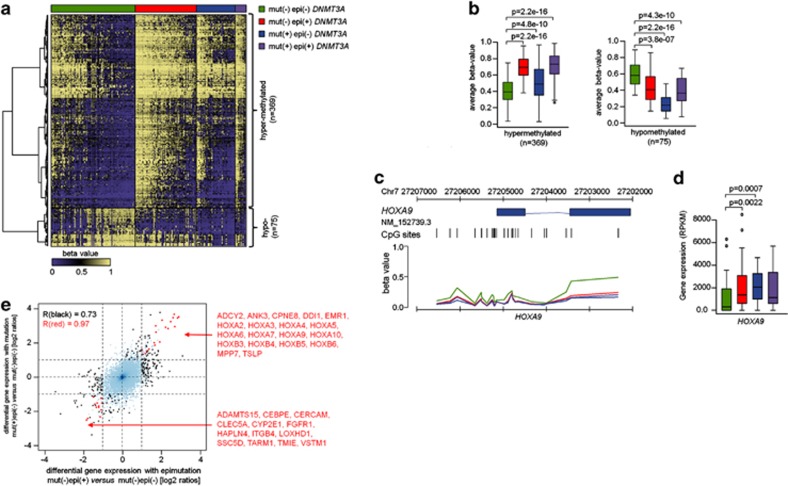
DNAm changes in samples with epimutation/mutation in *DNMT3A*. (**a**) Heatmap of 444 CpGs with significant DNAm changes between samples with epimutation and/or mutation in comparison with AML samples without such *DNMT3A* modifications (adjusted *P*-value <0.05; differential DNAm >20%). In particular, the DNAm pattern of hypomethylated CpGs was related in samples with either epimutation or mutation. (**b**) Average DNAm levels (*β*-values) of these CpG sites that are either hypermethylated or hypomethylated. (**c**) DNAm pattern of CpG sites related to the gene *HOXA9* is exemplarily depicted: AML samples with either mutations or epimutations in *DNMT3A* revealed significantly lower DNAm levels. (**d**) Conversely, *HOXA9* is significantly higher expressed in samples with *DNMT3A* mutation or epimutation. Statistical significance was estimated by Wilcoxon's rank-sum test. (**e**) Differential gene expression in AML samples with or without epimutation was plotted against differential gene expression in AML samples with or without genomic mutation in *DNMT3A*. Genes with differential expression in either one or both comparisons are highlighted in black and red, respectively. Gene IDs are provided for genes significant in both comparisons.

Next, we analyzed if the *DNMT3A* epimutation has a positive or negative correlation with DNAm changes:^9^ 3536 CpGs revealed significant DNAm changes (samples with *DNMT3A* mutation were excluded for this comparison; Supplementary Figure 12). Notably, almost the entire set (3523 CpGs) was hypermethylated in samples with *DNMT3A* epimutation. In contrast, comparison of DNAm profiles of AMLs with *DNMT3A* mutation *versus* wild-type AMLs (both without epimutation) revealed that only 78 CpGs were significantly hypermethylated, whereas 2595 CpGs were hypomethylated in mutated samples (Supplementary Figure 13) and many of them were associated with homeobox genes, including *HOXA2*, *A6*, *A7*, *A9*, *B2*, *B3*, *B4* and *B8.* The association of differential DNAm in samples with either mutation or epimutation was moderate, but overall there seemed to be a significant relationship (*P*=0.0005, *χ*^2^; Supplementary Figure 14). When epimutated and mutated samples were grouped together for comparison with samples without *DNMT3A* modifications, we detected 444 CpGs as differentially methylated (Figures 3a and b and Supplementary Table 4). These results revealed some concordance in DNAm changes upon either epimutation or mutation of *DNMT3A* and this was particularly observed in hypomethylated CpGs. Such overlapping hypomethylation was associated with several homeobox genes including in *HOXA9* (Figure 3c).

### Gene expression changes associated with *DNMT3A* epimutations

We have then analyzed if DNAm changes in AML with *DNMT3A* epimutation are also reflected on gene expression level. RNA-sequencing data from the TCGA^9^ revealed that 205 genes were differentially expressed in AML samples with or without *DNMT3A* epimutation (samples with *DNMT3A* mutation were excluded from this comparison): 81 genes were significantly downregulated, whereas 124 genes were upregulated, the latter including *HOXA1*, *A2*, *A3*, *A4*, *A5*, *A6*, *A7*, *A9*, *A10*, *B3*, *B4*, *B5* and *B6*. Chemical and genetic perturbations (CGP) demonstrated most significant association of upregulated genes with three data sets of AML mutations in *NPM1*^26, 27, 28^ (*P*-values always <10^−16^), which might point to an association of *DNMT3A* epimutation with *NPM1* mutation as mentioned above. Furthermore, differentially expressed genes were also found to be upregulated in hematopoietic stem and progenitor cells upon overexpression of NUP98-HOXA9 fusion protein (*P*<10^−16^).^29^ Heatmap analysis and principal component analysis supported the notion that differential gene expression in AML samples with epimutation is related to differential gene expression in samples with mutations in *DNMT3A* (Supplementary Figure 15). For example, *HOXA9* is significantly upregulated in both categories of *DNMT3A* modifications and this has been associated with poor prognosis in AML before (Figure 3d).^30^ In fact, almost the entire *HOXA* and *HOXB* cluster were significantly upregulated in AML samples with either epimutation or mutation in *DNMT3A.* The clear correlation of differentially expressed genes indicates that the molecular sequel of epimutation and mutation in *DNMT3A* might be related (Figure 3e). On the other hand, a significant compensatory upregulation in gene expression of *DNMT1*, *DNMT3B* or *DNMT3L* was not observed (Supplementary Figure 16).

### *DNMT3A* epimutations are associated with poor prognosis

**Figure 4:**
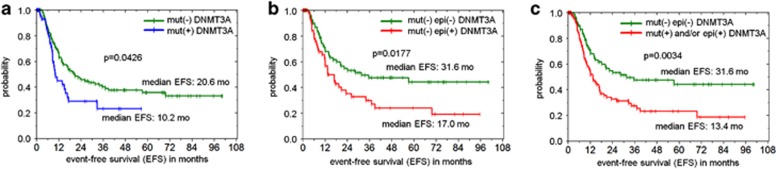
Kaplan–Meier analysis of event-free survival (EFS) of individuals with AML. (**a**) Patients with *DNMT3A* mutations (mut+) reveal a significantly shorter EFS than patients without mutation (mut−) in AML patients from TCGA (*n*=194).^9^ (**b**) In analogy, patients with epimutation (epi+) revealed a significantly shorter EFS than those without epimutation (epi− samples with *DNMT3A* mutation were excluded from this analysis; *n*=145). (**c**) Results became even more significant when patients with normal *DNMT3A* status (mut− and epi−) were compared with those with mutation and/or epimutation (*n*=194).

Finally, we analyzed the impact of *DNMT3A* epimutations on clinical prognosis. Indeed, hypermethylation within the gene *DNMT3A* was associated with significantly shorter event-free survival (Figure 4) and shorter OS (Supplementary Figure 17) in the data set of TCGA.^9^ However, such a pronounced effect could not be recapitulated in our own data set, which can likely be attributed to the relatively small set of 88 AML samples. Furthermore, it is not clear if the prognostic relevance is independent of cytogenetic risk analysis: when we only considered samples from TCGA with intermediate- or poor-risk score, the results were no more significant (Supplementary Figure 17e). Thus, larger data sets might be required to determine whether or not epimutations in *DNMT3A* have independent diagnostic relevance. Either way, the results support the notion that epimutations in *DNMT3A*, just as genomic mutations,^8, 12, 31^are rather associated with poor-risk score and poor prognosis in AML.

## Discussion

Epimutations define heritable changes in gene activity, which, unlike classical gene mutations, are not due to changes in DNA sequence.^32, 33^ They occur in somatic cells, particularly in tumor suppressor genes.^34^ *RB* methylation was identified as the first example of epimutation^35^ and subsequently many other oncogenes have been shown to be methylated in sporadic cancers.^36, 37, 38^ In this study, we describe that AML patients often reveal aberrant hypermethylation in *DNMT3A* and the same effect was also observed in AML cell lines. We used an absolute threshold of 10% DNAm level to select patients with epimutations—this threshold was not reached in controls and other authors used even lower cutoffs to define epimutations.^39^ On the other hand, microarray data as well as bisulfite pyrosequencing indicated that the methylation level was sometimes even above 50%. With regard to the heterogeneous composition of malignant and normal cells, this indicates that the epimutation is homozygous in these cases. This might either be due to uniparental disomy, selection pressure or to a related upstream mechanism that entails the epimutation on both alleles of *DNMT3A*. Notably, very high aberrant DNA hypermethylation was only observed in AML samples, but not in chronic myeloid leukemia or other myeloproliferative neoplasms. It might be a common phenomenon that epimutations are disease specific if the corresponding genomic mutations are specifically enriched in this disease, too.

We have recently analyzed DNAm changes upon culture expansion of hematopoietic stem and progenitor cells *in vitro*:^21^ the same genomic region of *DNMT3A* as described in this study was hardly methylated in freshly isolated CD34+ cells from cord blood but it was significantly hypermethylated in culture-expanded CD34+cells (*P*<10^−46^) and this was associated with downregulation of transcripts 2 and 4. Transcript 4 does not possess methyltransferase activity, but downregulation may still be functionally relevant if it contributes to regulatory complexes. This led us to the assumption that variant transcripts of *DNMT3A* are important for regulation of self-renewal *versus* differentiation. Spliceosome assembly occurs at the same time as transcription and therefore the DNA structure can directly influence alternative splicing. It has been shown that a DNA-binding protein, CCCTC-binding factor (CTCF), can promote inclusion of weak upstream exons by mediating local RNA polymerase II pausing.^14^ Notably, a CTCF binding site is also located within DMR2 of *DNMT3A* and hence the epimutation may be relevant for regulation of alternative splicing. In fact, the epimutation is associated with moderate downregulation of transcripts 2 and 4 when considering the relative expression towards transcript 1. These results indicate that the epimutation has impact on expression of variant transcripts but it does not necessarily indicate functional competition of corresponding proteins. Unexpectedly, a much more pronounced downregulation of transcript 2 was observed in samples with genomic mutations in *DNMT3A*. It is conceivable that mutations as well as DNAm changes influence alternative splicing and/or the stability of *DNMT3A* transcript 2.^14, 15^ Either way, it is striking that epimutations and mutations of *DNMT3A* seem to have similar consequences on the relative expression of this transcript. So far, there is little knowledge about the specific functions of variant transcripts, their stability and their impact on the DNAm profile—yet, it is well possible that the various isoforms of *DNMT3A* have different sequence specificity or interact with different partners.

Loss of DNAm activity of mutated *DNMT3A* has been demonstrated before,^11, 40^ whereas hypermethylation upon epimutation of *DNMT3A* appears to be paradoxical. This might be due to the fact that the mutations within the methyltransferase domain have impact on all active transcripts, whereas the epimutations rather influence relative expression—and potentially alternative splicing—of specific transcripts. On the other hand, hypermethylation has also been described in inducible conditional *Dnmt3a*-knockout mice, indicating that dysregulation of this methyltransferase causes widespread indirect changes.^7^ The opposite effects of epimutations and mutations on DNAm changes did not refer to the same CpG sites. Overall, there was even a moderate association in DNAm changes. Furthermore, there was a clear correlation of differentially expressed genes. Other groups have previously demonstrated multiple expression clusters associated with *DNMT3A* mutations but none of them were clearly defined by the mutation status.^8, 11, 31^ Our results now suggest that this might be attributed to epimutations in *DNMT3A* conferring similar changes in gene expression pattern. Extensive upregulation of *HOXA7* and *HOXB2*, *B3*, *B4*, *B5*, *B6*, *B7* and *B8* has been demonstrated before in AML samples with mutation in *DNMT3A*.^11^ In analogy, many genes of the *HOXA* and *HOXB* cluster were upregulated in samples with an epimutation in *DNMT3A*. *HOX* genes have a crucial role in regulation of normal hematopoiesis and they appear to be involved in pathogenesis of AML and other cancers.^41, 42, 43^ It is therefore conceivable that the molecular sequel of *DNMT3A* mutations/epimutations on regulation of *HOX* genes have a central role for disease progression.

The DNAm level at DMR2 was significantly higher in AML samples without epimutation, but both modifications were significantly associated with mutations of *IDH1*, *IDH2*, *RUNX1* and *NPM1.* Epimutations and mutations in *DNMT3A* were enriched in AML samples with an intermediate or poor cytogenetic risk. Wakita and co-workers^44^ have recently suggested that an initial mutation in an epigenetic-modifying gene—such as *DNMT3A*—may entail genetic instability leading to resistance to therapy and relapse.^44^ A similar effect might also apply for epimutations. Furthermore, analysis of TCGA data revealed that both of them are associated with significantly shorter event-free survival. These findings further substantiate the perception that *DNMT3A* epimutations mimic genetic mutations of *DNMT3A*, and they suggest that both mutations synergize with *IDH1/2* mutations, *RUNX1* mutations and *NPM1* mutations to alter DNAm, histone modification, hematopoietic differentiation and cell survival.^24^ It is conceivable that *DNMT3A* modifications represent early events—potentially even initiating events—that then entail the less frequent mutations in *IDH1*, *IDH2*, *RUNX1* and *NPM1*. However, this hierarchy of clonal development needs to be further analyzed. On the other hand, epimutations and mutations in *DNMT3A* were not observed in core-binding factor-AMLs and hardly in acute promyelocytic leukemias and this supports the notion that these entities have different pathophysiologic origin.^25, 45^

Analysis of the epimutation in *DNMT3A* is based on DNAm at a single CpG site—yet, differential DNAm at neighboring CpGs within the CPI revealed a very high correlation. Considering that bisulfite pyrosequencing is a relatively inexpensive high-throughput technique and that our assay is site specific, screening of larger patient populations is feasible. This approach might be useful for risk stratification and it might also be relevant for therapeutic decisions: cytosine nucleoside analogs—such as azacitidine and decitabine—are approved for the treatment of AML. They are incorporated into the DNA and bind covalently to catalytic sites of all DNMTs.^46^ It is conceivable that DNMT inhibitors are particularly effective in patients with epimutations in *DNMT3A* as they reflect aberrant DNA hypermethylation at many CpG sites. Furthermore, it has been demonstrated that in patients with genomic mutations in *DNMT3A* the relative resistance to chemotherapy can be overcome with high-dose daunorubicin treatment, which then improved survival rates.^47^ It needs to be assessed if the epimutation in *DNMT3A* has a significant effect on the outcome with dose-intensive chemotherapy, too.

The observation that about half of the AML patients either have a mutation or an epimutation in *DNMT3A* indicates a high relevance for disease development. Despite the very different nature of these epigenetic or genetic aberrations, their molecular and functional sequels appear to be related. The bisulfite pyrosequencing assay described here provides a relatively simple and cost-effective approach to screen for aberrant DNA hypermethylation within *DNMT3A*. The impact for diagnosis, risk stratification, disease monitoring and choice of therapeutic regimens needs to be analyzed further in independent data sets—yet both mutations and epimutations in *DNMT3A* should both be taken into account.
