# Supplementary material for: Epimutations mimic genomic mutations of DNMT3A in acute myeloid leukemia
Source: Leukemia. 2013 Dec 20;28(6):1227–34. doi: 10.1038/leu.2013.362 (PMC4051212; doi:10.1038/leu.2013.362)
Supplement: Supplementary Information [file leu2013362x2.doc]

# Supplemental Information

# Epimutations Mimic Genomic Mutations of *DNMT3A* in Acute Myeloid Leukemia

Edgar Jost^1,*^, Qiong Lin^2,*^, Carola Ingrid Weidner^3^, Stefan Wilop^1^, Melanie Hoffmann^1^, Thomas Walenda^3^, Mirle Schemionek^1^, Oliver Herrmann^1^, Martin Zenke^2^, Tim Henrik Brümmendorf^1^, Steffen Koschmieder^1^ and Wolfgang Wagner^3^

^1^ Department of Oncology, Hematology and Stem Cell Transplantation, RWTH Aachen University Medical School, Aachen, Germany; ^2^ Institute for Biomedical Engineering - Cell Biology, RWTH Aachen University Medical School, Aachen, Germany; ^3^ Helmholtz-Institute for Biomedical Engineering, Stem Cell Biology and Cellular Engineering, RWTH Aachen University Medical School, Aachen, Germany; * These authors contributed equally to this work

**Table of Content**

[Supplemental Figure 1: Pie diagram for the interplay of three DMRs in *DNMT3A*. 2](#__RefHeading___Toc369862591)

[Supplemental Figure 2: DMRs of *DNMT3A* in hematopoietic cell types and malignant cell lines. 2](#__RefHeading___Toc369862592)

[Supplemental Figure 3: Bisulfite pyrosequencing assays of DMR2. 3](#__RefHeading___Toc369862593)

[Supplemental Figure 4: Bisulfite pyrosequencing results of individual CpG sites. 4](#__RefHeading___Toc369862594)

[Supplemental Figure 5: Correlation of DNAm within the CpG island of DMR2. 4](#__RefHeading___Toc369862595)

[Supplemental Figure 6: Association of DNAm at DMR2 with gender and age. 5](#__RefHeading___Toc369862596)

[Supplemental Figure 7: DNAm in relation to blast counts. 5](#__RefHeading___Toc369862597)

[Supplemental Figure 8: Mutational analysis of the *DNMT3A* hotspot region. 6](#__RefHeading___Toc369862598)

[Supplemental Figure 9: Hypomethylation at DMR1 is associated with good cytogenetic risk. 6](#__RefHeading___Toc369862599)

[Supplemental Figure 10: Organization of mutations in *DNMT3A* categories*.* 7](#__RefHeading___Toc369862600)

[Supplemental Figure 11: Expression of *DNMT3A* transcripts. 8](#__RefHeading___Toc369862601)

[Supplemental Figure 12: DNAm changes in AML with epimutation in *DNMT3A*. 9](#__RefHeading___Toc369862602)

[Supplemental Figure 13: DNAm changes in AML with mutation in *DNMT3A*. 10](#__RefHeading___Toc369862603)

[Supplemental Figure 14: Correlation of differential DNAm in patients with either epimutation or mutation. 10](#__RefHeading___Toc369862604)

[Supplemental Figure 15: Gene expression in AML with epimutation in *DNMT3A*. 11](#__RefHeading___Toc369862605)

[Supplemental Figure 16: Gene expression of *DNMT1, DNMT3B* and *DNMT3L* is not affected. 11](#__RefHeading___Toc369862606)

[Supplemental Figure 17: Overall survival among AML patients with *DNMT3A* epimutation. 12](#__RefHeading___Toc369862607)

[Supplemental Table 1: Characteristics of the patient cohort. 13](#__RefHeading___Toc369862608)

[Supplemental Table 2: Primers for Pyrosequencing. 13](#__RefHeading___Toc369862609)

[Supplemental Table 3: Primers for qRT-PCR. 14](#__RefHeading___Toc369862610)

[Supplemental Table 4: CpG sites with DNAm changes in epimutated and mutated AMLs. 14](#__RefHeading___Toc369862611)

[References of supplemental data 14](#__RefHeading___Toc369862612)

###
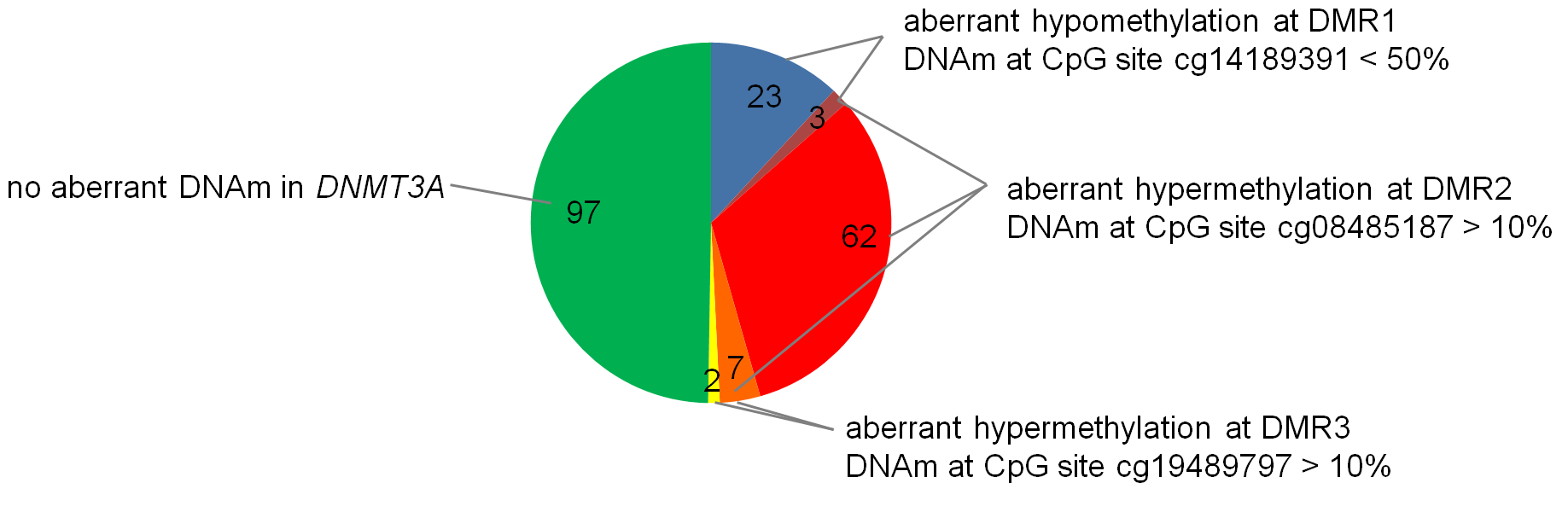


### Supplemental Figure 1: Pie diagram for the interplay of three DMRs in *DNMT3A*.

DNA-methylation profiles of AML samples from the TCGA repository data^1^ revealed three differentially methylated regions (DMRs). Based on DNAm in normal blood^2^ we defined cutoffs for aberrant DNAm: DMR1, particularly at CpG site cg14189391, was always methylated more than 50% in normal blood,^2^ whereas it was hypomethylated (DNAm < 50%) in 26 AML samples. In contrast DMR2 and DMR3 were non-methylated in normal blood^2^ (particularly at CpG sites cg08485187 and cg19489797), whereas they were hypermethylated (DNAm > 10%) in 72 and 9 AML samples, respectively. Notably, overlapping hypomethylation at DMR1 and hypermethylation at DMRs 2 or 3 was underrepresented (p = 0.0038 and p = 0.61, respectively; two-sided Fisher’s exact test), whereas hypermethylation correlated at DMR2 and DMR3 (p = 0.013).


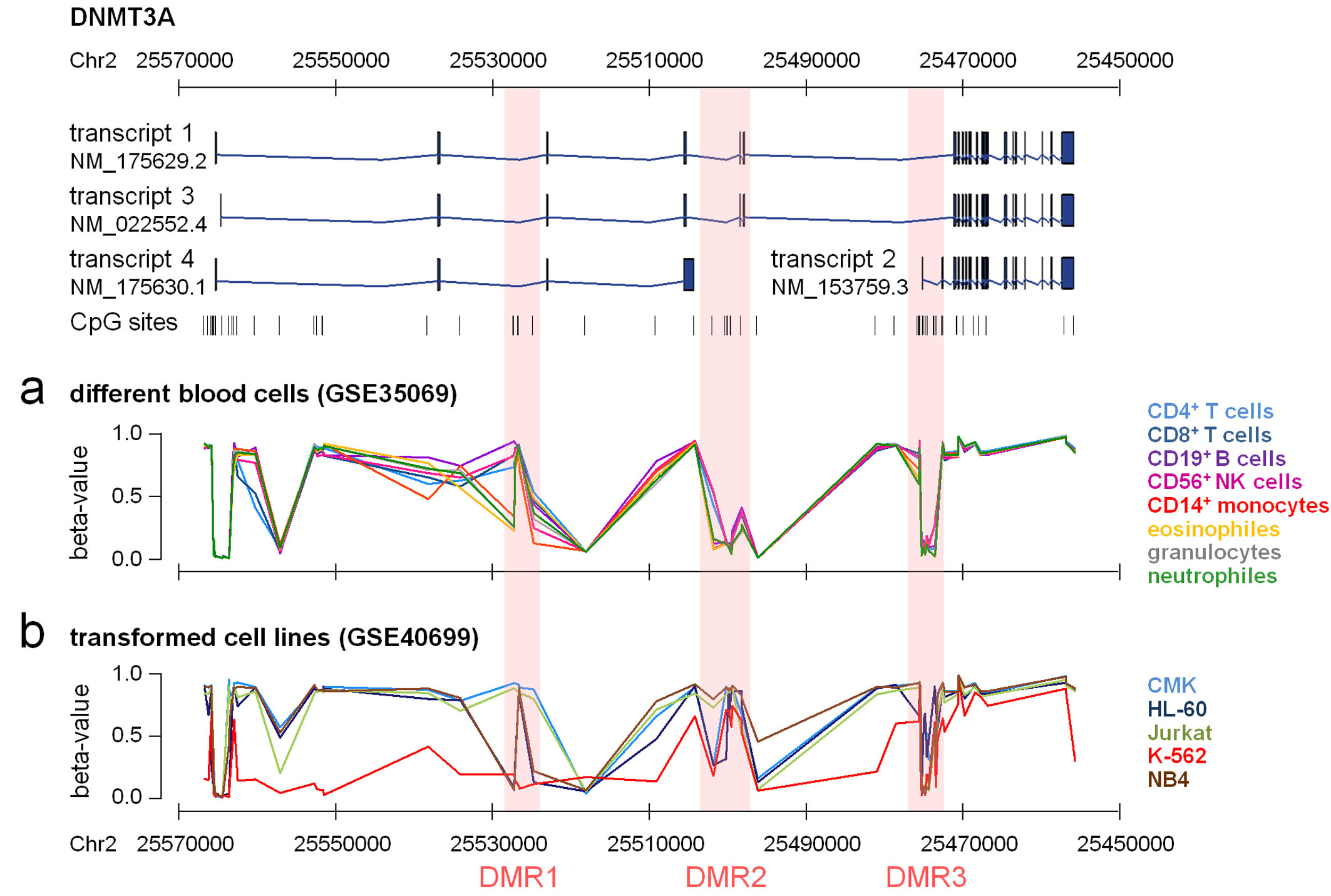


### Supplemental Figure 2: DMRs of *DNMT3A* in hematopoietic cell types and malignant cell lines.

**(a)** Beta-values of CpG sites within *DNMT3A* were analyzed in DNAm profiles of various different cell types (Th cells = CD4^+^ T cells, Tc cells = CD8^+^ T cells, NK cells = CD56^+^ NK cells, B cells = CD19^+^ B cells, monocytes = CD14^+^ monocytes, eosinophiles, granulophiles, and neutrophiles).^3^ DNAm at DMR1 revealed some variation between different cell types and it appeared to be hypomethylated particularly in myeloid cells – with may coincide with hypomethylation in AML. However, DMR2 and DMR3 were rather non-methylated irrespective of the corresponding cell type. **(b)** In contrast, DNAm profiles of malignant cell lines (GSE40699, generated by ENCODE) revealed aberrant hypermethylation particularly at DMR2.


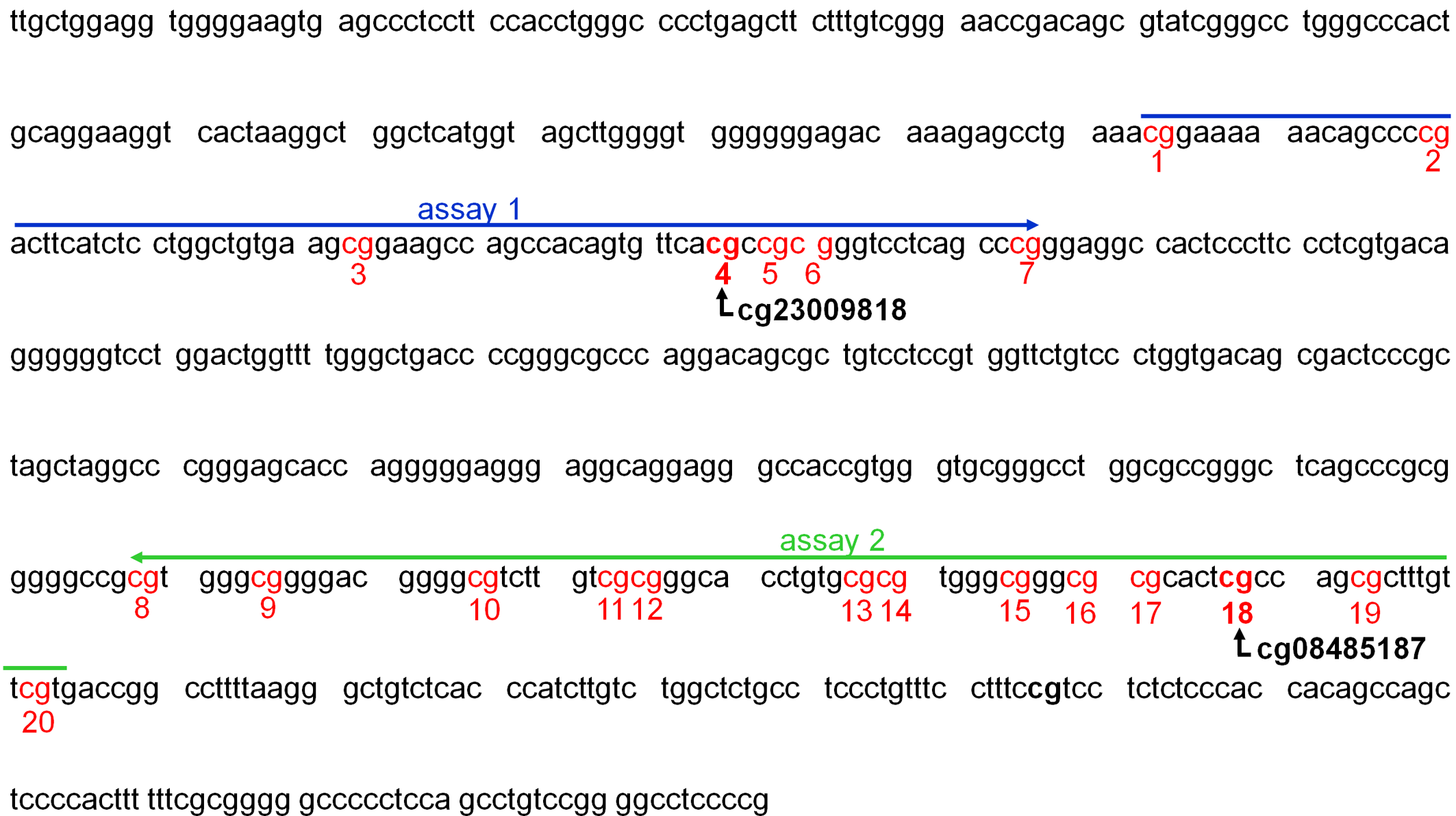


### Supplemental Figure 3: Bisulfite pyrosequencing assays of DMR2.

DNAm was analyzed in blood samples using two different pyrosequencing assays. CpG sites which are covered by these assays are enumerated in red. These assays comprise two CpG sites which are also addressed by probe sets of the Illumina HumanMethylation450k BeadChip (cg23009818 and cg08485187).


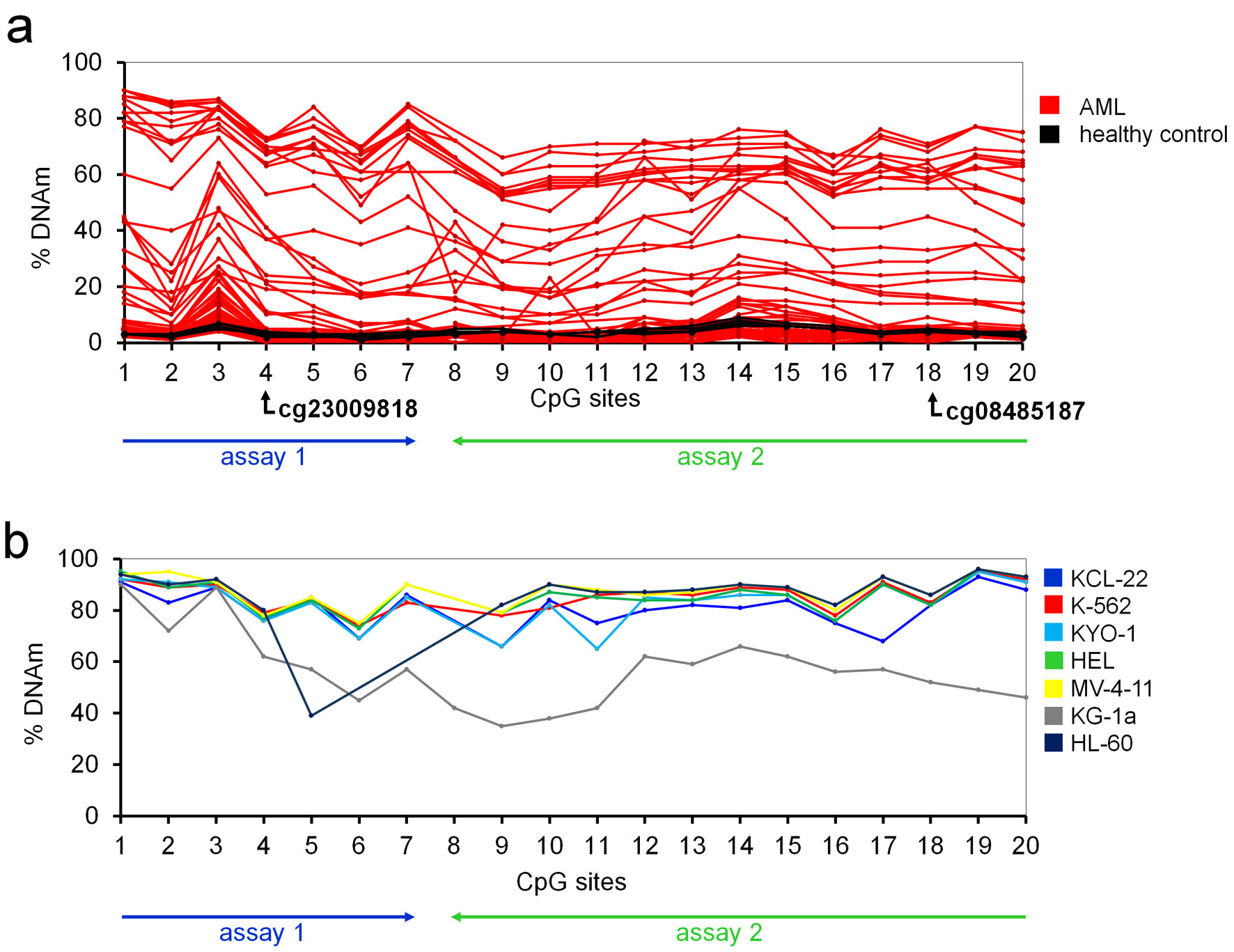


### Supplemental Figure 4: Bisulfite pyrosequencing results of individual CpG sites.

**(a)** Blood from healthy controls (n = 26) and from AML patients (n = 88) were analyzed with the two pyrosequencing assays. DNAm was always below 10% in all healthy controls and at all CpG sites. DNA-hypermethylation in AML samples was not restricted to individual CpGs but appeared to be relatively constant throughout the whole DMR. **(b)** Hypermethylation was also observed in various cell lines derived from AML (KCL-22, K-562, KYO-1, HEL, MV-4-11, KG-1a, and HL-60).


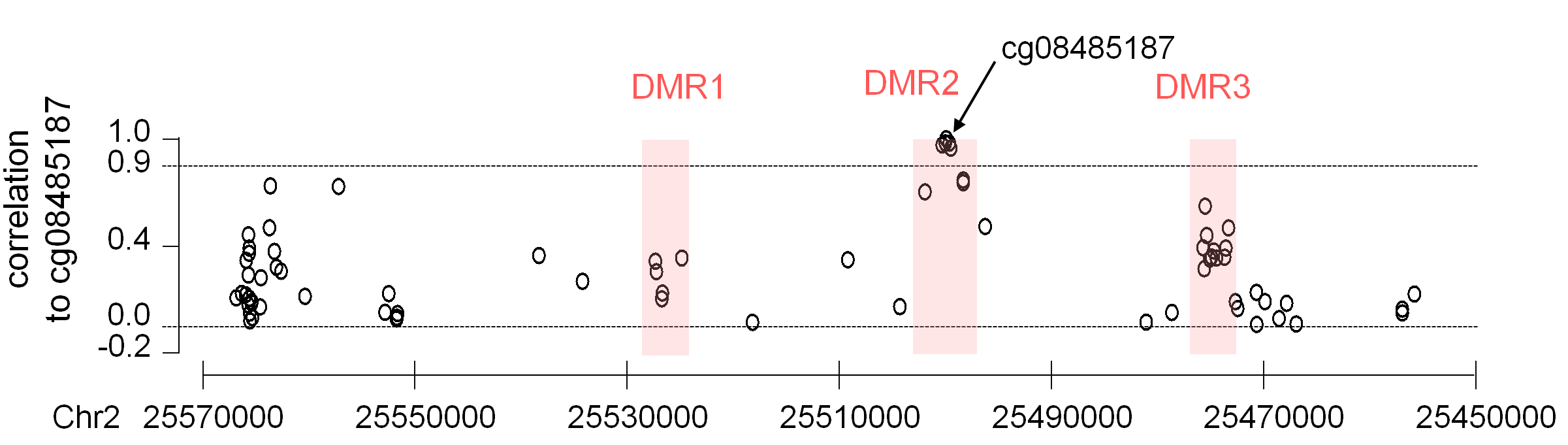


### Supplemental Figure 5: Correlation of DNAm within the CpG island of DMR2.

Besides the CpG site cg08485187, three other probe sets were located in the same CGI of DMR2. All of them showed very high correlation coefficients with DNAm at cg08485187 in TCGA data (cg23009818: R=0.976; cg13558695: R=0.977; cg08493294: R=0.965). In addition, cg17742416, which is in the shore region, is also highly correlated (R=0.948). DNAm of all 20 CpGs covered by the pyrosequencing assays revealed a correlation of more than 0.95, too (data not shown). Therefore, DNAm at cg08485187 is a good surrogate for overall methylation of the CpG island (CpG:_61) in DMR2.


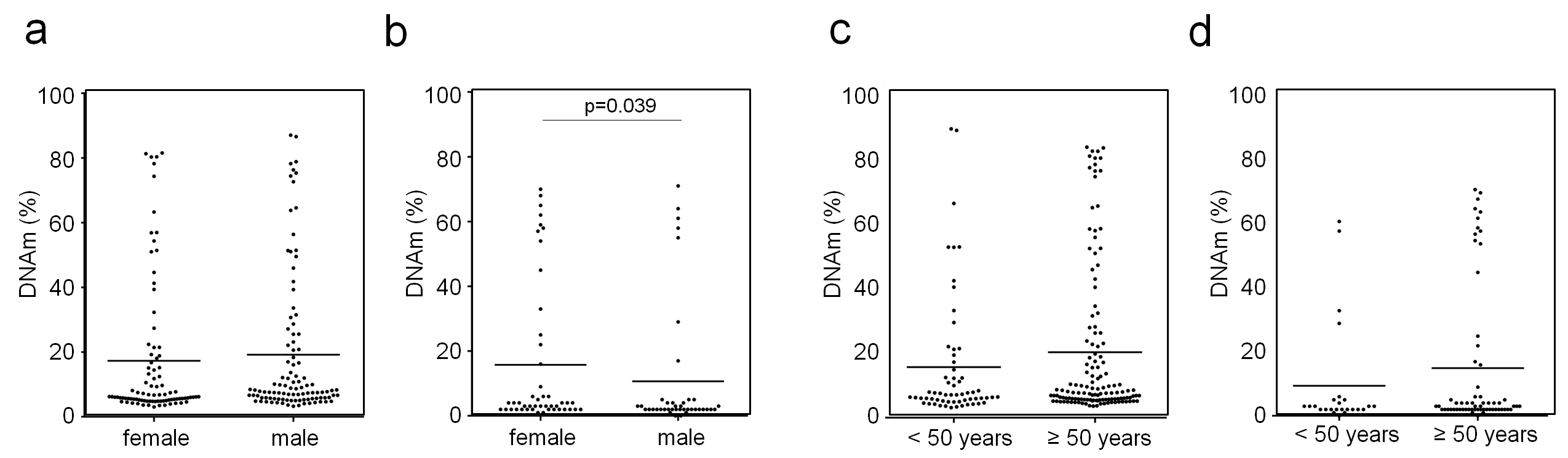


### Supplemental Figure 6: Association of DNAm at DMR2 with gender and age.

DNAm values at cg08485187 was analyzed in TCGA repository data^1^ **(a,c)** and in our pyrosequencing data **(b,d)**. There was not clear association with gender in TCGA data **(a)** and it was only slightly increased in our female samples **(b)**. In tendency, DNAm at DMR2 was higher in samples of elderly donors but this was not significant **(c,d)**. Statistical significance was estimated by Wilcoxon rank-sum test.


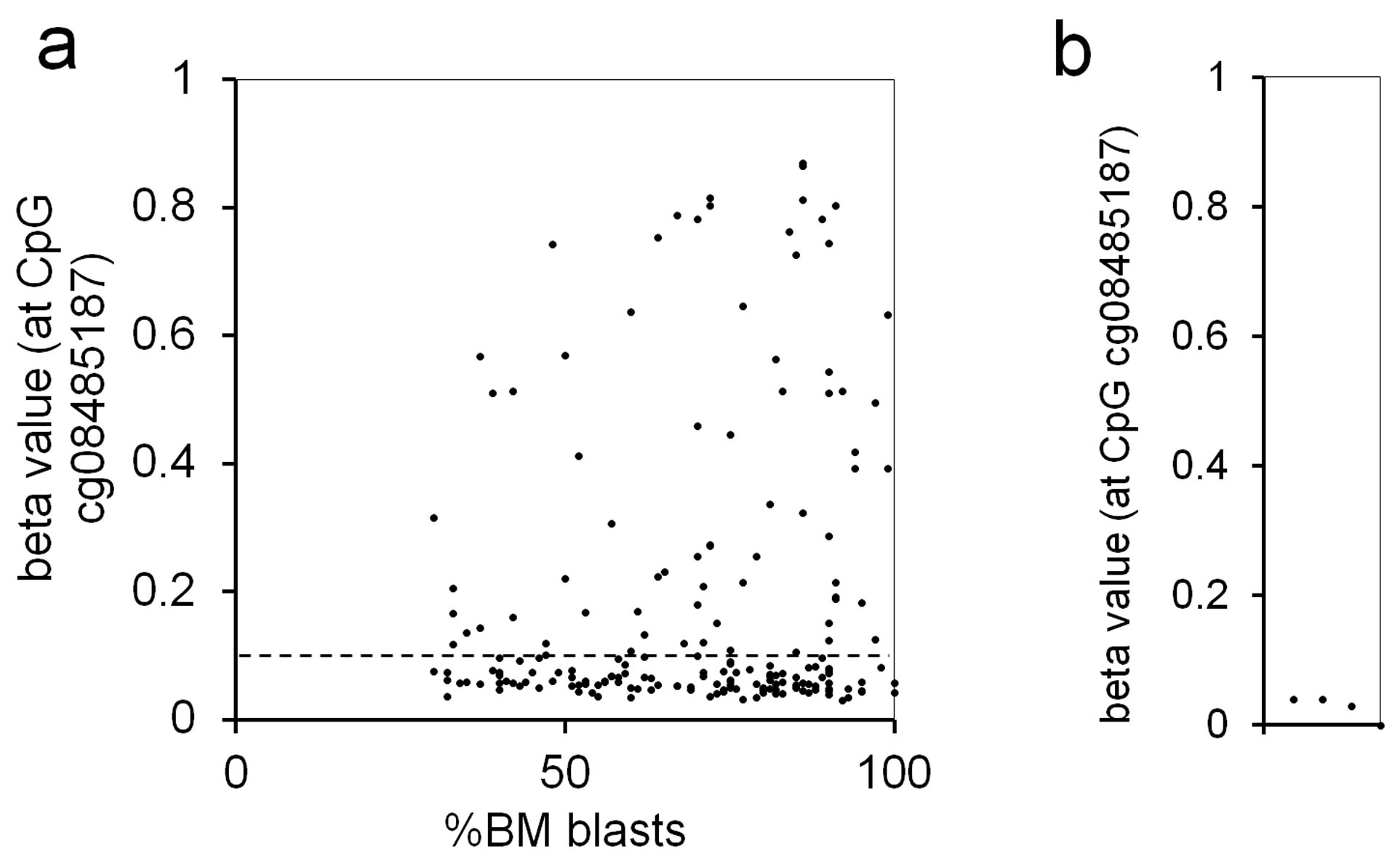


### Supplemental Figure 7: DNAm in relation to blast counts.

**(a)** The DNA methylation level at the relevant CpG site (cg08485187) is plotted against blast counts of the TCGA repository data.^1^ The dotted line indicates 10% DNAm cutoff which has been used to discern samples with epimutation. There is no linear relationship between aberrant DNAm and blast counts. The high DNAm levels in several samples indicate that the epimutation affects both alleles. **(b)** For additional control, we have analyzed DNAm by pyrosequencing in four stem cell harvests obtained by leukapheresis. Despite high content of stem and progenitor cells the DNAm level was always low.


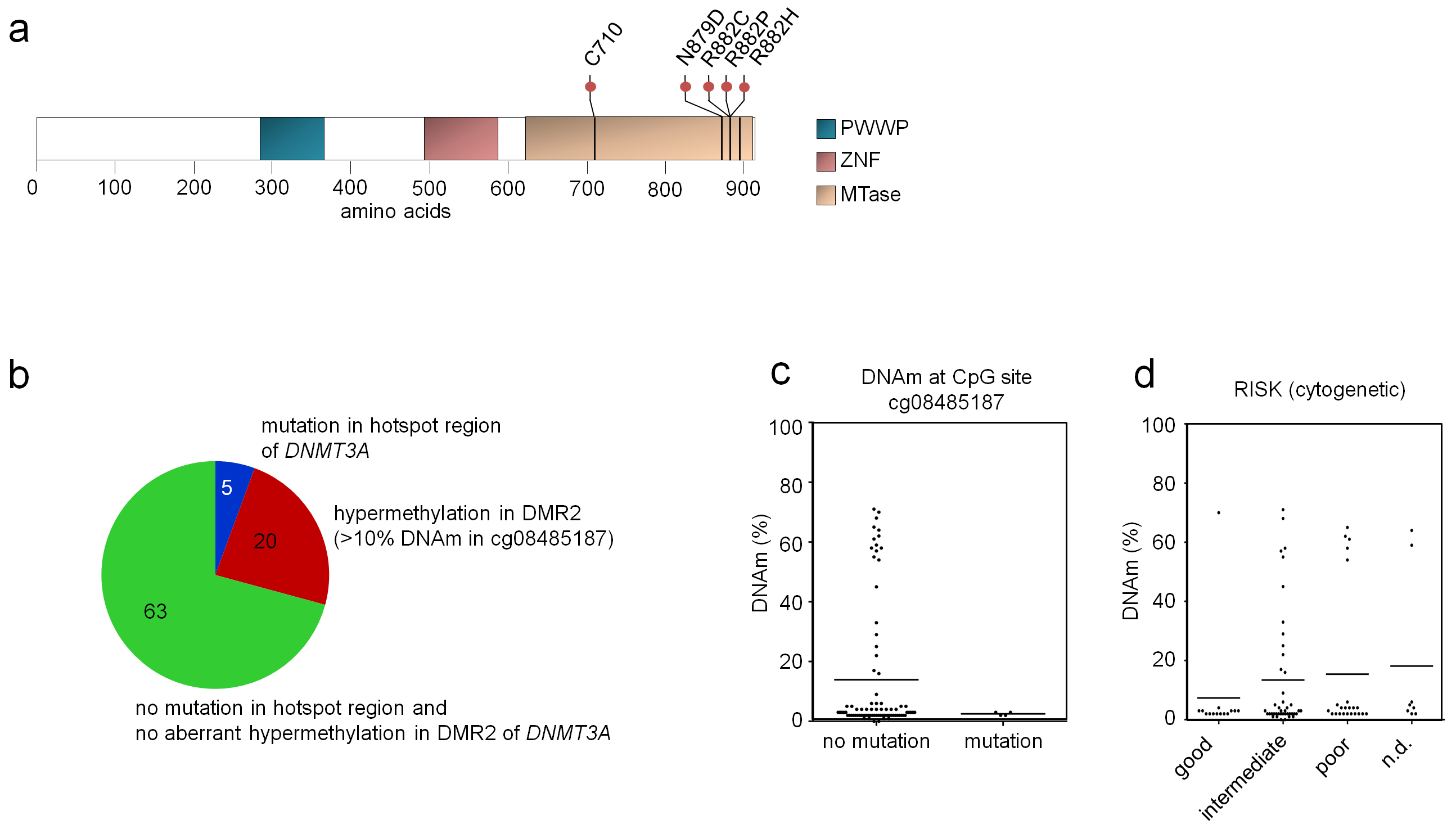


### Supplemental Figure 8: Mutational analysis of the *DNMT3A* hotspot region.

**(a)** Schematic presentation of DNMT3A. The conserved proline-tryptophan-tryptophan-proline (PWWP), zinc finger (ZNF), and methyltransferase (MTase) domains are shown. In our exploratory analysis we sequenced exons 18, 19, and 23. These assays account for 70% of the genomic mutations identified in TCGA repository data. In three AML patients, we observed mutations at the hotspot region (R882), one sample revealed a so far non-described mutation in close vicinity (N879), and one patient revealed a stop mutation in exon 18 (C710). Sequencing of HL-60, HEL, KG-1a, MV-4-11, NALM6, K-562, and KCL-22 cell lines did not reveal mutations at that region (data not shown). DNAm was only analyzed in DMR2 by bisulfite pyrosequencing. **(b)** Pie diagram of AML patients with mutation and epimutation (DNAm level at cg08485187 > 10% as determined by bisulfite pyrosequencing). **(c,d)** Epimutations were only found in samples without mutation at the frequently mutated regions (not significant; p = 0.58, two-sided Fisher’s exact test) and they were hardly associated with good cytogenetic risk score (not significant; p = 0.29; n.d. = not determined).


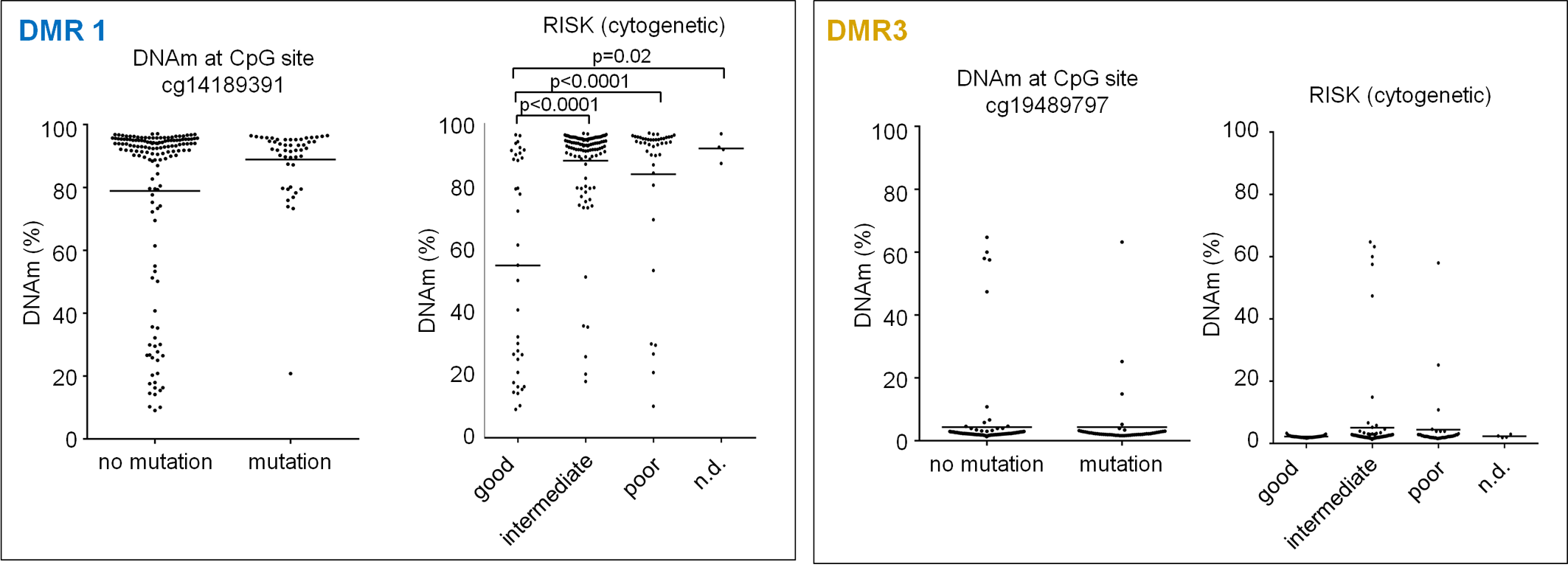


### Supplemental Figure 9: Hypomethylation at DMR1 is associated with good cytogenetic risk.

Analysis of DNAm profiles of AML samples in conjunction with mutation status of *DNMT3A* and cytogenetic risk score^1^ revealed that aberrant hypomethylation of DMR1 was rather observed in AML without mutation in *DNMT3A* – in analogy to aberrant hypermethylation of DMR2. However, DNAm at DMR1 was significantly lower in samples with good cytogenetic risk score whereas hypermethylation in DMR3 occurred rather in AML with intermediate or poor risk (n.d. = not determined).


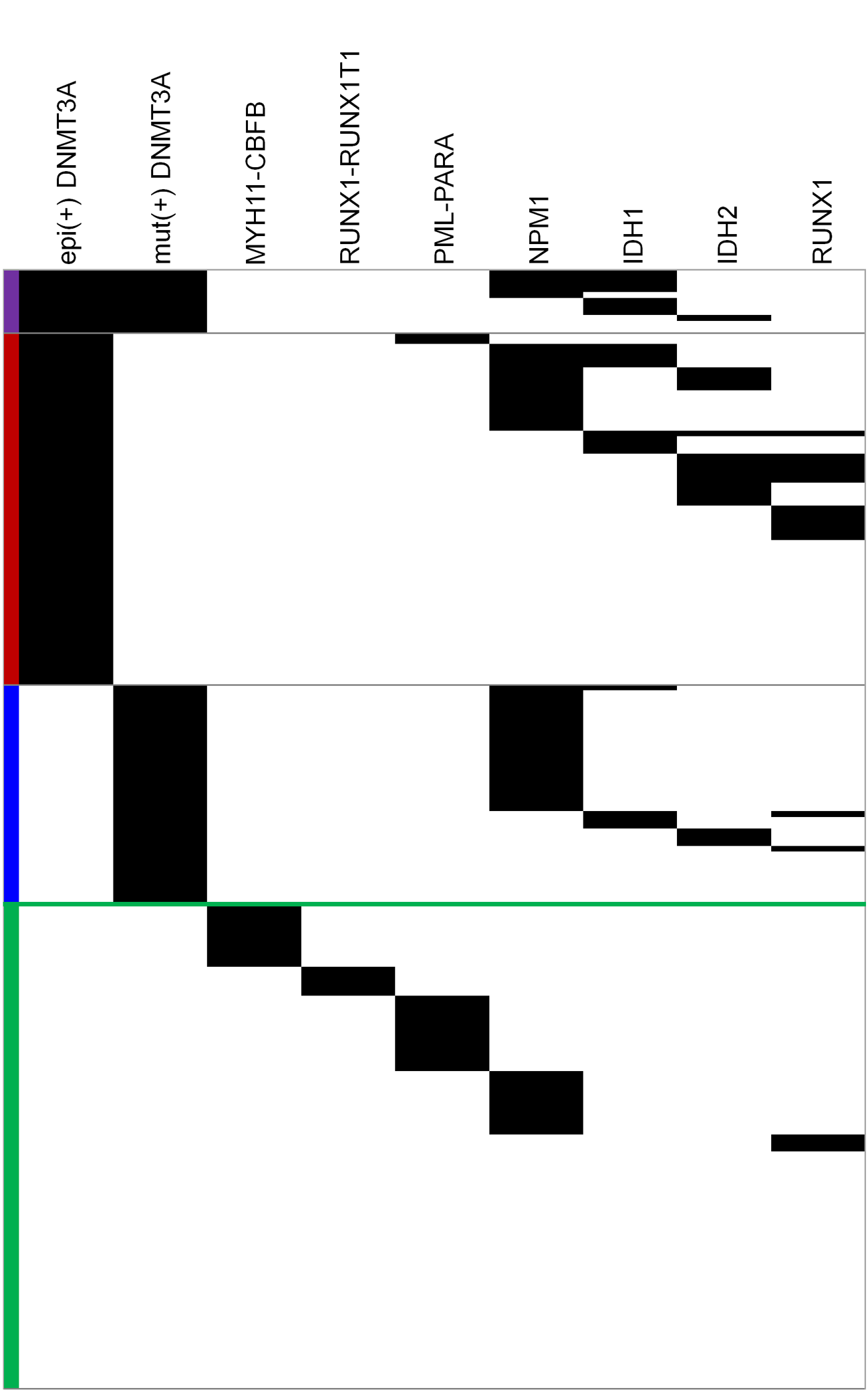


### Supplemental Figure 10: Organization of mutations in *DNMT3A* categories*.*

AML samples of TCGA have been analyzed using either whole-genome sequencing (50 cases) or whole-exome sequencing (150 cases).^1^ 194 samples with DNAm profiles were subsequently grouped in those with epimutation (epi+) and/or mutation (mut+) in *DNMT3A* and analyzed with regard to gene fusions or mutations. A selection of relevant mutations is presented which revealed differences in samples with *DNMT3A* modifications (black depicts mutation or epimutation of corresponding genes). For clarity, other mutations are not presented. Notably, *MYH11-CBFB, RUNX1-RUNX1T1* and *PML-PARA* fusions were significantly enriched in epi(-)mut(-) samples (p < 0.001, p = 0.009, and p < 0.001, respectively; chi-squared test) – these fusions have been associated with good prognosis.^4^ In contrast, the frequent mutations in *NPM1, IDH1, IDH2* and *RUNX1* were significantly enriched in patients with mutations and/or epimutation in *DNMT3A* (p < 0.001, p < 0.001, p < 0.001, and p < 0.027, respectively; the color code on the left corresponds to *DNMT3A* modifications according to Figure 2A).


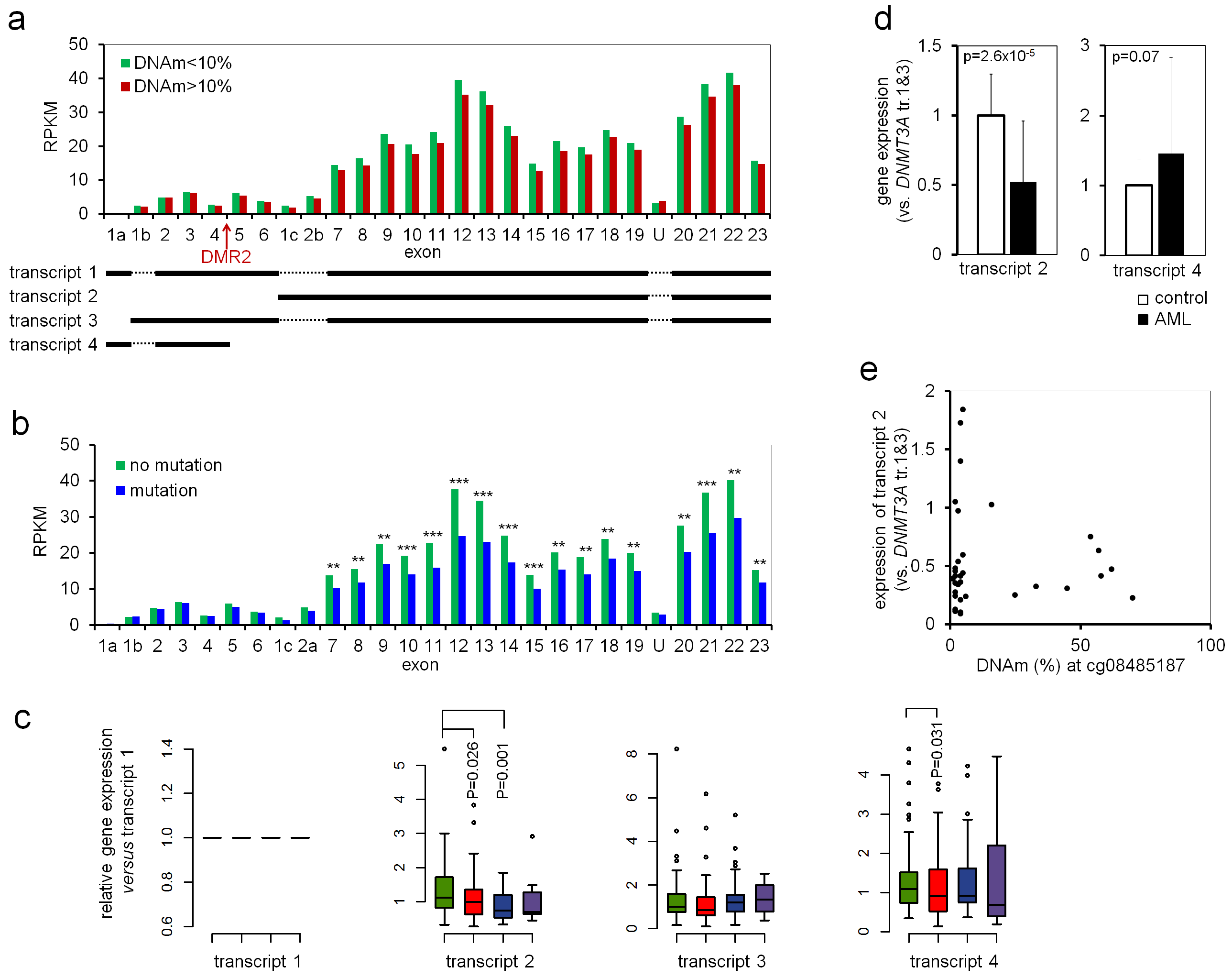


### Supplemental Figure 11: Expression of *DNMT3A* transcripts.

**(a)** Transcription of exons were analyzed in RNA-sequencing data of TCGA.^1^ The scheme depicts which exons are comprised in the four main transcripts of *DNMT3A* (alternative exons included in variant transcripts are indicated by letters; U = undefined transcript association). In samples with aberrant hypermethylation in DMR2 (DNAm > 10% at cg08485187) particularly exons involved in transcript 2 were slightly less expressed than in patients without epimutation (RPKM = reads per kilobase per million reads). The same tendency was also observed in microarray data (data not shown).^1^ **(b)** Notably, these exons revealed an even more pronounced down-regulation in samples with *DNMT3A* mutation (** p < 0.01; *** p < 0.001; two-sided t test). **(c)** To further analyze if the epimutation and mutation have impact on the relative expression of variant transcripts we used quantile normalization to scale the expression levels of four transcripts to same level. Expression levels of individual transcripts were then normalized to transcript 1. The analysis revealed that transcript 2 and transcript 4 are relatively less expressed in AML with epimutation in *DNMT3A* (Wilcoxson rank-sum test). **(d)** qRT-PCR analysis of variant transcripts in our AML samples was hampered by the high variation of housekeeping genes (e.g. *GAPDH*) between AML samples (n = 18) and healthy controls (n = 34). Therefore, we normalized towards expression levels of transcript 1&3 (primer pairs do not discern between transcript 1 and 3). Overall, transcript 2 of *DNMT3A* appeared to be less expressed in AML samples than in healthy controls. In contrast, transcript 4 appeared to be slightly increased in AML samples. **(e)** High levels of DNAm at DMR2 were rather associated with low expression of transcript 2. This trend is in accordance with TCGA data, but it did not reach significance level.


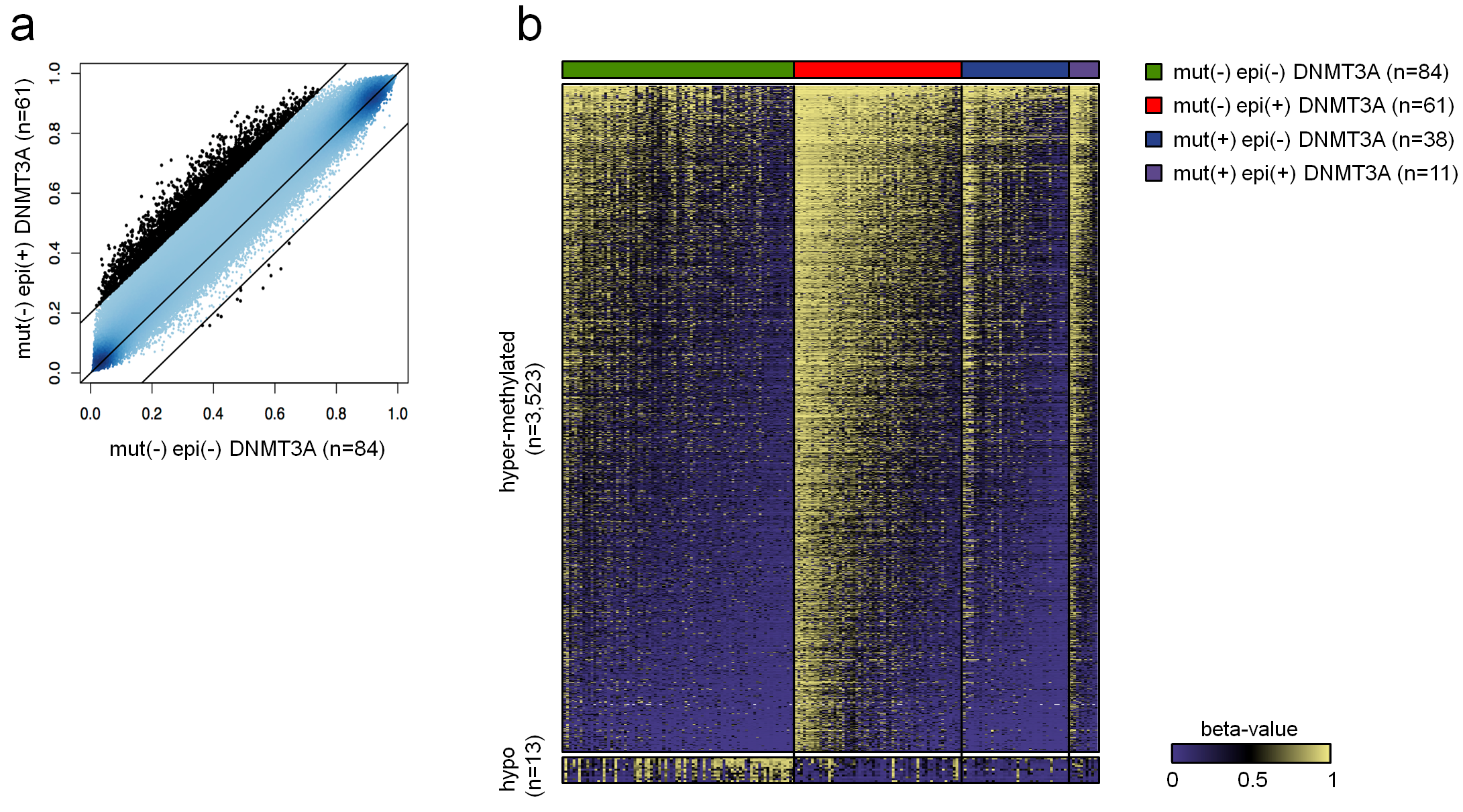


### Supplemental Figure 12: DNAm changes in AML with epimutation in *DNMT3A*.

DNAm profiles of TCGA^1^ were compared between samples with or without epimutation (mutated samples were excluded from this analysis; mut-). **(a)** Scatter-plot analysis reveals that epimutation (epi+) is associated with significant hypermethylation of many CpGs (adjusted p-value < 0.05; DNAm changes > 20%; depicted in black). **(b)** Heat-map presentation of the corresponding 3,536 CpGs. Notably, the few hypomethylated CpGs revealed a similar pattern also in samples with *DNMT3A* mutations.


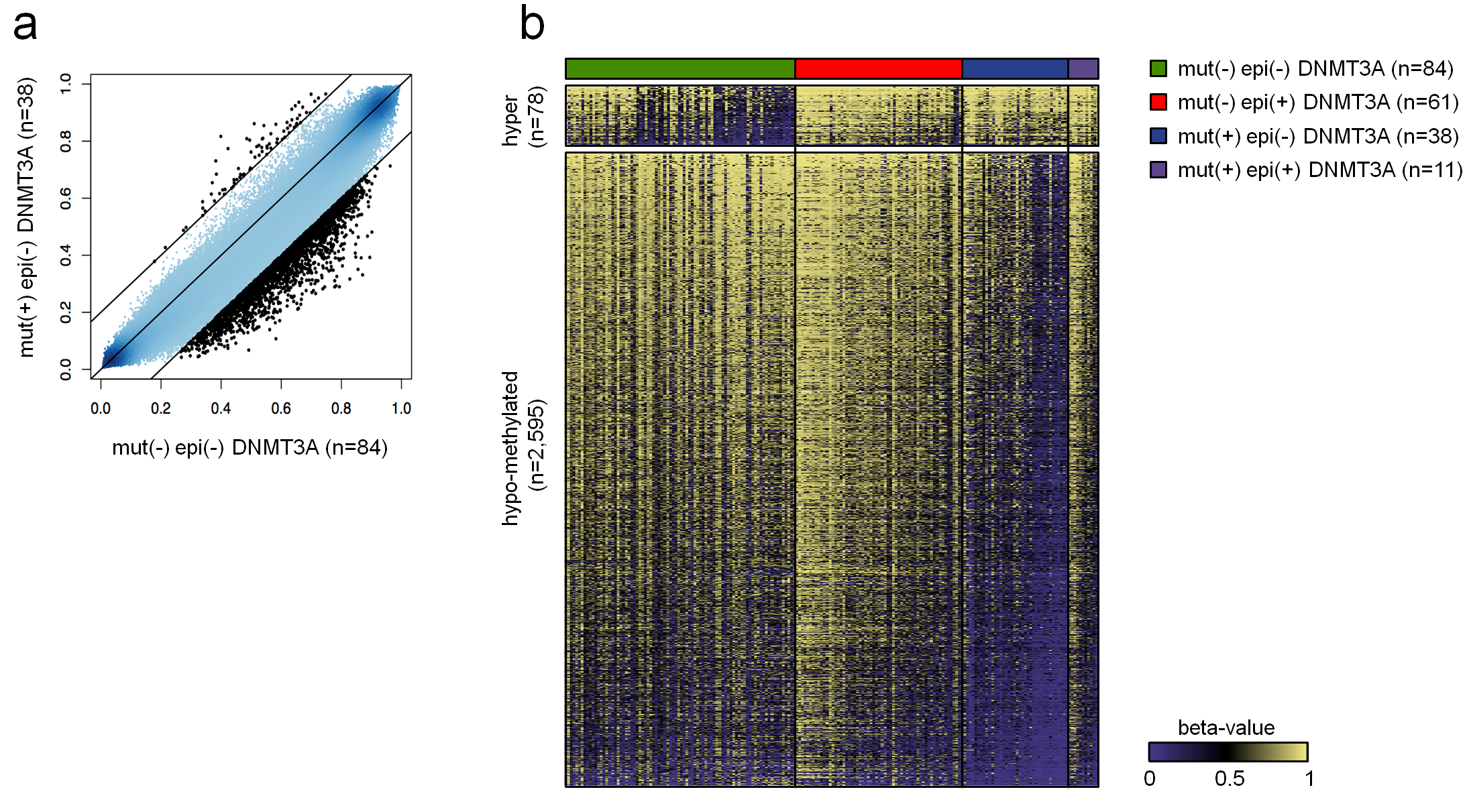


### Supplemental Figure 13: DNAm changes in AML with mutation in *DNMT3A*.

DNAm profiles of TCGA^1^ were compared between samples with or without mutation (mut+/-). Samples with epimutation were excluded from this analysis (epi-). **(a)** The scatter-plot reveals that mutations in *DNMT3A* are associated with significant hypomethylation of many CpGs (adjusted p-value < 0.05; DNAm changes > 20%; depicted in black). **(b)** Heat-map presentation of the corresponding 2,673 CpGs. Notably, the few hypermethylated CpGs revealed a similar pattern also in samples with *DNMT3A* epimutations.


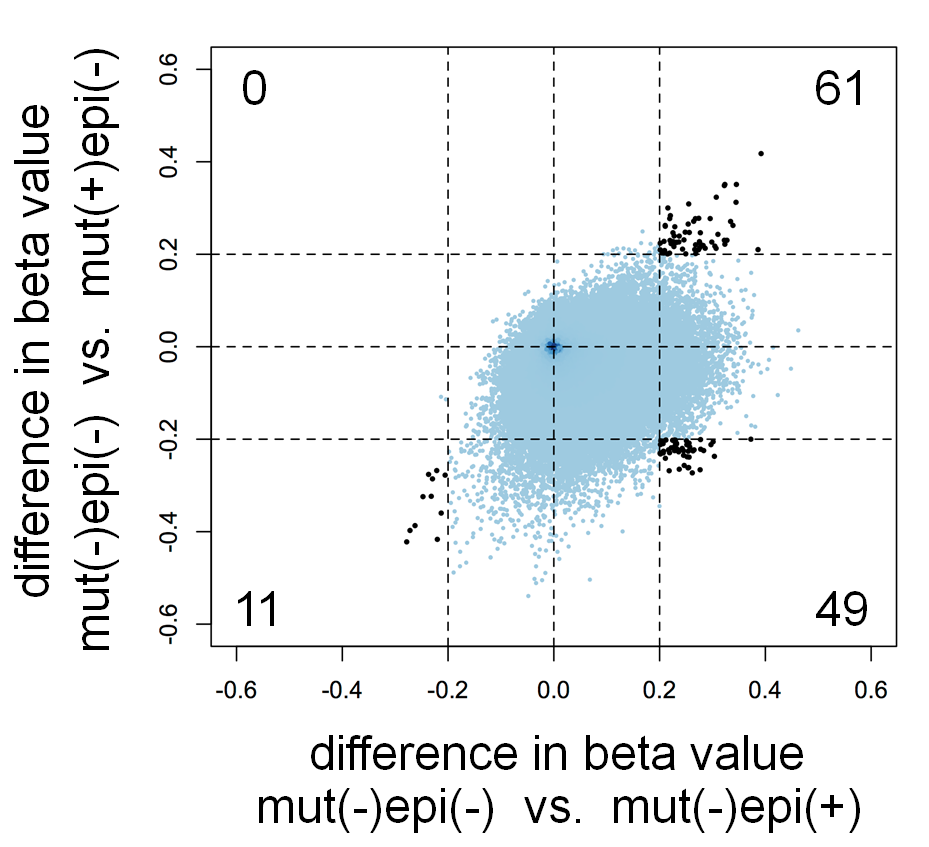


### Supplemental Figure 14: Correlation of differential DNAm in patients with either epimutation or mutation.

Differential DNAm levels in patients with or without epimutation (mut-epi- *versus* mut-epi+) were plotted against differential DNAm levels in patients with or without mutation (mut-epi- *versus* mut+epi-). CpG sites with differential DNAm of more than 20% in both comparisons are depicted in black. Chi-square analysis of these CpG sites indicated that there is a significant relationship between the two comparisons (p = 0.0005).


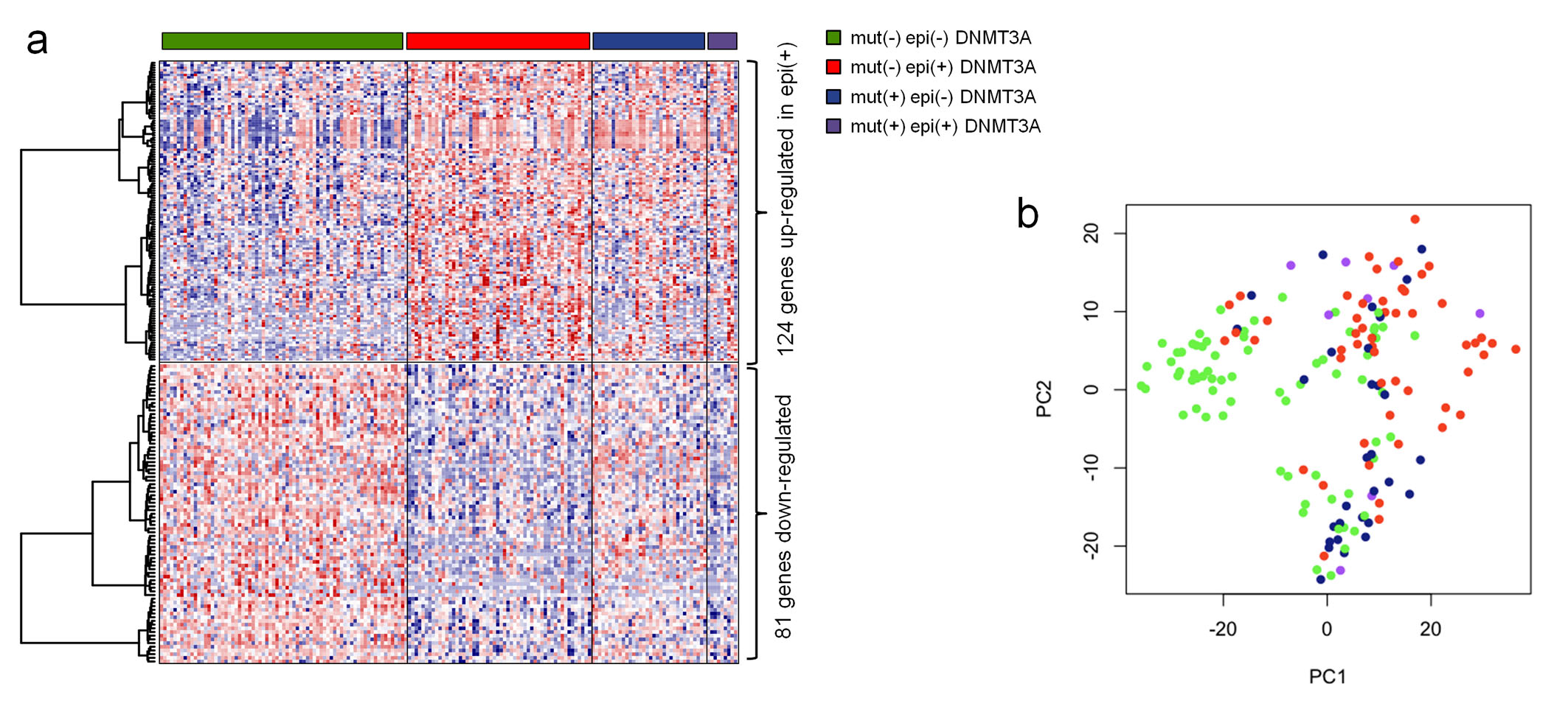


### Supplemental Figure 15: Gene expression in AML with epimutation in *DNMT3A*.

**(a)** RNA-sequencing data from TCGA^1^ were compared in samples with or without epimutation (mutated samples were excluded from this analysis). The heatmap depicts 205 differentially expressed genes. **(b)** PCA analysis of these genes demonstrated that AML samples with mutation in *DNMT3A* were rather related to samples with epimutation in *DNMT3A* (PC1 and PC2 = principal component 1 and 2).


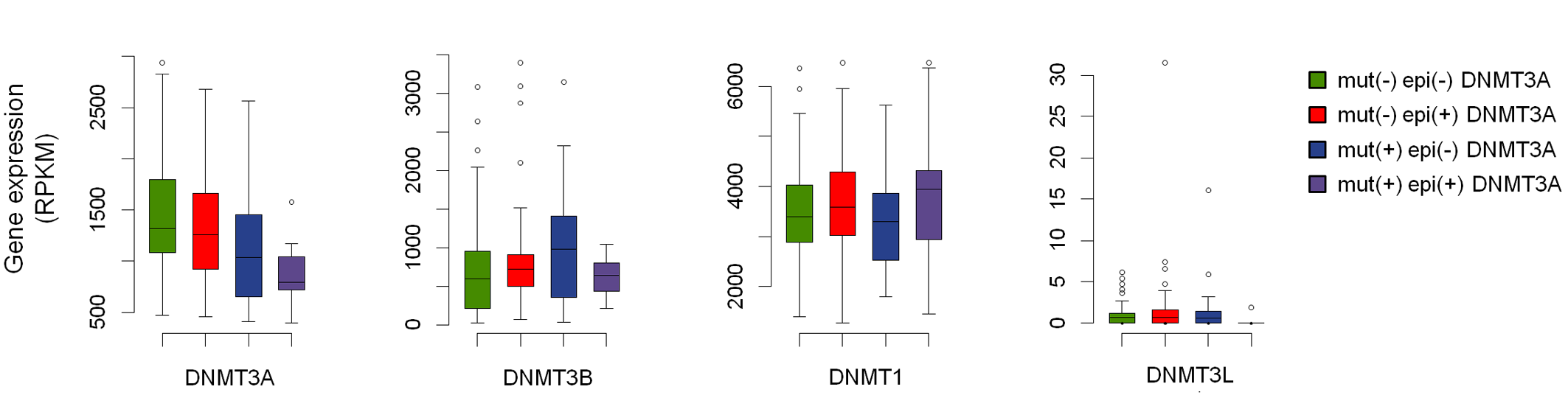


### Supplemental Figure 16: Gene expression of *DNMT1, DNMT3B* and *DNMT3L* is not affected.

Given that AML samples with epimutations revealed extensive DNA-hypermethylation, it might be anticipated that gene expression of other methyltransferases has compensatory effects. However, this was not observed in RNA-sequencing data from TCGA^1^ (results were not significant).


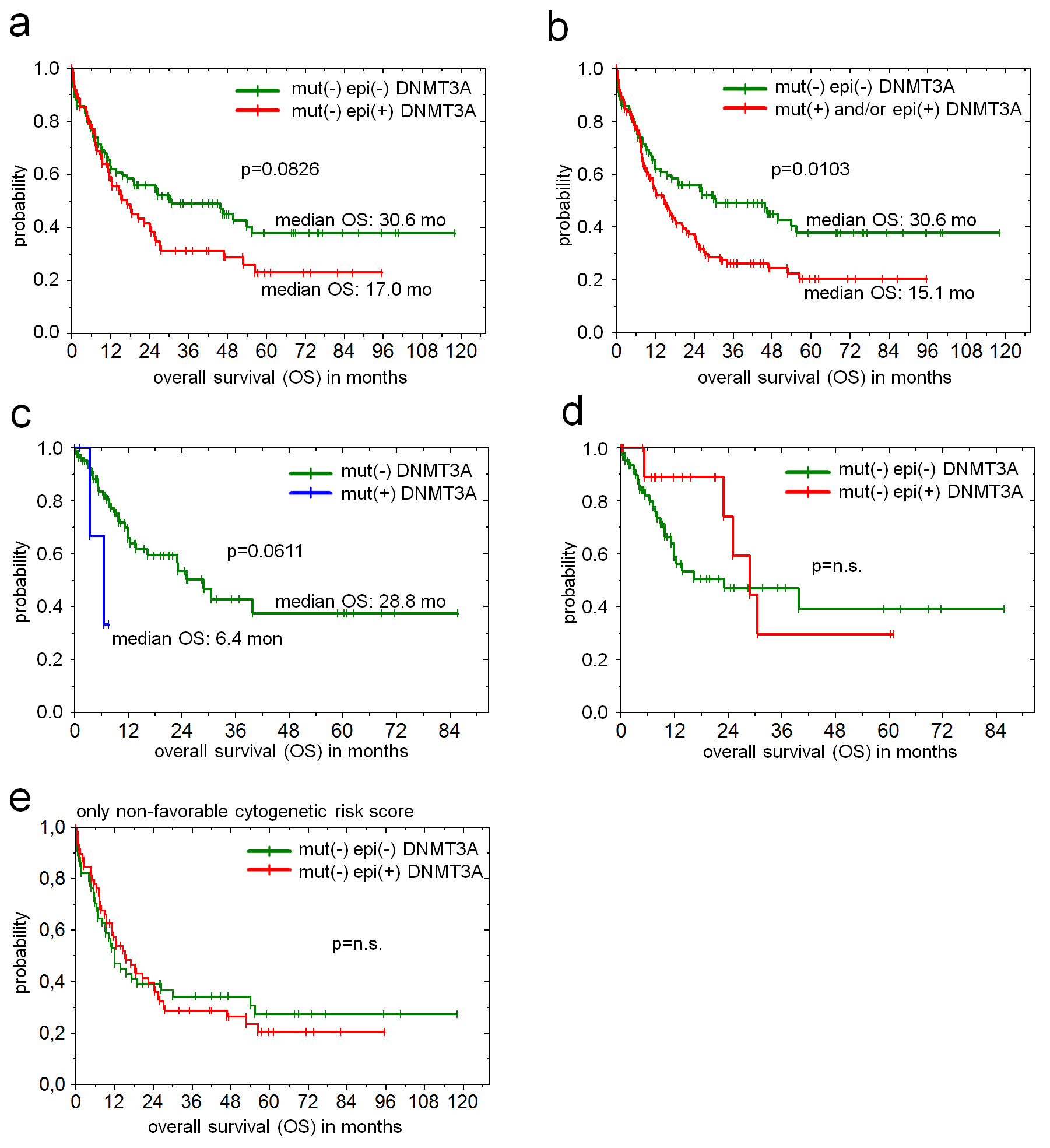


### Supplemental Figure 17: Overall survival among AML patients with *DNMT3A* epimutation.

**(a)** Patients with *DNMT3A* epimutations (epi+) reveal shorter OS than patients without epimutation (epi-; samples with mutation were excluded from this analysis) in data from TCGA^1^ although results did not reach statistical significance (n = 145). **(b)** Comparison of patients with mutation and/or epimutation with patients without such *DNMT3A* modification revealed significant impact on overall survival (n = 194). **(c)** In our dataset of 88 AMLs the OS was shorter in patients with *DNMT3A* mutation - in analogy to previous publications.^5-8^ **(d)** A significant effect of the epimutation on OS was not observed in our dataset (n.s. = not significant). **(e)** Multivariate analysis of OS in AML samples of TCGA with either intermediate or poor cytogenetic risk score did not reveal significant effects – therefore, it is yet unclear if the epimutation in *DNMT3A* is an independent parameter for prognosis in AML.

### Supplemental Table 1: Characteristics of the patient cohort.

| **Parameter** | **amount** |
| --- | --- |
| number of patient | 88 |
| age, years (median and range) | 58 (18 - 89) |
| gender |  |
| male | 40 |
| female | 48 |
| FAB subtype |  |
| M0 | 12 |
| M1 | 24 |
| M2 | 12 |
| M3 | 3 |
| M4 | 29 |
| M4Eo | 3 |
| M5 | 5 |
| karyotype |  |
| favorable (CBF leukemia) | 14 |
| intermediate | 43 |
| adverse | 23 |
| no data | 8 |
| laboratory parameters (median and range) |  |
| WBC (G/l) | 30.5 (0.8 – 294.6) |
| hemoglobin (g/l) | 96.2 (68 - 163) |
| platelet count (G/l) | 118 (7 - 782) |
| LDH (UI/l) | 642 (124 - 7662) |
| preexisting myelodysplastic syndrome | 19 |
| induction chemotherapy | 61 |

FAB, French-American-British; CBF, core binding factor;

WBC, white blood cell; LDH, lactate dehydrogenase

### Supplemental Table 2: Primers for Pyrosequencing.

| **Primer** | **Sequence** |
| --- | --- |
| assay 1 forward | 5`-GGTTTGGGTTTATTGTAGGAAGGTTATTAAGGT-3´ |
| assay 1 reverse | 5`-AATCCAAAACCCCCCTATCACGAAA-Bio-3´ |
| assay 1 sequencing | 5`-GTGGGGGGAGATAAA-3´ |
| assay 2 forward | 5`-Bio-TTAGGTTCGGGAGTATTAGGGGGAGG-3´ |
| assay 2 reverse | 5`-AACAAAACCAAACAAAATAAATAAAACAACCC-3´ |
| assay 2 sequencing | 5`-AAATAAAACAACCCTTAAAA-3´ |

### Supplemental Table 3: Primers for qRT-PCR.

| **Primer** | **Sequence** |
| --- | --- |
| DNMT3A tr. 1&3 forward | 5`-ACCCTGCCTGAAGCCTCAAG-3´ |
| DNMT3A tr. 1&3 reverse | 5`-AAGGTGAGCCTCGGCATGG-3´ |
| DNMT3A tr. 2 forward | 5`-GTGGATCGTAGCCTGAAAG-3´ |
| DNMT3A tr. 2 reverse | 5`-TGGTGGCATTCTTGTCC-3´ |
| DNMT3A tr. 4 forward | 5`-AAGCGGGTGAGTCCTCAGC-3´ |
| DNMT3A tr. 4 reverse | 5`-CATATGCGCAGGCTGCATCC-3´ |
| GAPDH forward | 5`-TTCGTCATGGGTGTGAAC-3´ |
| GAPDH reverse | 5`-CTGTGGTCATGAGTCCTT-3´ |

### Supplemental Table 4: CpG sites with DNAm changes in epimutated and mutated AMLs.

This EXCEL table gives additional information for 444 CpG sites related to the heatmap of Figure 3A. IDs for probe sets, gene names, relation to genomic regions and CpG islands, DNAm changes and adjusted p-value, as well as mean DNAm levels for the each four different categories (epi+/-; mut+/-) are presented. This table is provided as separate file.

### References of supplemental data

(1) Ley TJ, Miller C, Ding L, Raphael BJ, Mungall AJ, Robertson AG, et al. Genomic and epigenomic landscapes of adult de novo acute myeloid leukemia. *N Engl J Med* 2013; **368**: 2059-74.

(2) Hannum G, Guinney J, Zhao L, Zhang L, Hughes G, Sadda S, et al. Genome-wide Methylation Profiles Reveal Quantitative Views of Human Aging Rates. *Mol Cell* 2013; **49**: 459-367.

(3) Reinius LE, Acevedo N, Joerink M, Pershagen G, Dahlen SE, Greco D, et al. Differential DNA methylation in purified human blood cells: implications for cell lineage and studies on disease susceptibility. *PLoS ONE* 2012; **7**: e41361.

(4) Jourdan E, Boissel N, Chevret S, Delabesse E, Renneville A, Cornillet P, et al. Prospective evaluation of gene mutations and minimal residual disease in patients with core binding factor acute myeloid leukemia. *Blood* 2013; **121**: 2213-2223.

(5) Marcucci G, Metzeler KH, Schwind S, Becker H, Maharry K, Mrozek K, et al. Age-related prognostic impact of different types of DNMT3A mutations in adults with primary cytogenetically normal acute myeloid leukemia. *J Clin Oncol* 2012; **30**: 742-750.

(6) Ley TJ, Ding L, Walter MJ, McLellan MD, Lamprecht T, Larson DE, et al. DNMT3A mutations in acute myeloid leukemia. *N Engl J Med* 2010; **363**: 2424-2433.

(7) Yamashita Y, Yuan J, Suetake I, Suzuki H, Ishikawa Y, Choi YL, et al. Array-based genomic resequencing of human leukemia. *Oncogene* 2010; **29**: 3723-3731.

(8) Ribeiro AF, Pratcorona M, Erpelinck-Verschueren C, Rockova V, Sanders M, Abbas S, et al. Mutant DNMT3A: a marker of poor prognosis in acute myeloid leukemia. *Blood* 2012; **119**: 5824-5831.
